# Overview of Liquid Sample Preparation Techniques for Analysis, Using Metal-Organic Frameworks as Sorbents

**DOI:** 10.3390/molecules29194752

**Published:** 2024-10-08

**Authors:** Jakub Woźniak, Jakub Nawała, Daniel Dziedzic, Stanisław Popiel

**Affiliations:** Faculty of Advanced Technologies and Chemistry, Institute of Chemistry, Military University of Technology, Kaliskiego Str. 2, 00-908 Warsaw, Poland; jakub.wozniak.116@gmail.com (J.W.); jakub.nawala@wat.edu.pl (J.N.); daniel.dziedzic@wat.edu.pl (D.D.)

**Keywords:** metal-organic frameworks (MOFs), solid-phase extraction (SPE), solid-phase microextraction (SPME), micro-solid-phase extraction (µ-SPE), magnetic solid-phase extraction (MSPE), pipette-tip solid-phase extraction (PT-SPE), matrix solid-phase dispersion (MSPD), stir-bar sorptive extraction (SBSE)

## Abstract

The preparation of samples for instrumental analysis is the most essential and time-consuming stage of the entire analytical process; it also has the greatest impact on the analysis results. Concentrating the sample, changing its matrix, and removing interferents are often necessary. Techniques for preparing samples for analysis are constantly being developed and modified to meet new challenges, facilitate work, and enable the determination of analytes in the most comprehensive concentration range possible. This paper focuses on using metal-organic frameworks (MOFs) as sorbents in the most popular techniques for preparing liquid samples for analysis, based on liquid-solid extraction. An increase in interest in MOFs-type materials has been observed for about 20 years, mainly due to their sorption properties, resulting, among others, from the high specific surface area, tunable pore size, and the theoretically wide possibility of their modification. This paper presents certain advantages and disadvantages of the most popular sample preparation techniques based on liquid-solid extraction, the newest trends in the application of MOFs as sorbents in those techniques, and, most importantly, presents the reader with a summary, which a specific technique and MOF for the desired application. To make a tailor-made and well-informed choice as to the extraction technique.

## 1. Introduction

The development of analytical chemistry is constantly observed, among others, in the field of improving the techniques of sample preparation for analysis. Samples delivered to the laboratory are often characterized by a complex matrix, in which, in addition to interesting analytes, there is often a significant number of interferents hindering the analysis. In addition, the analysis is often restrained by the small amount of sample provided and the low concentration of analytes contained in it. It is often necessary to change the sample matrix so that it can be analyzed using the appropriate technique. The primary role of sample preparation for analysis is usually concentration, the isolation of analytes from the sample matrix, and the removal of some or all interferents, often changing the sample matrix or transforming analytes into appropriate derivatives that allow their detection (derivatization) [1]. A big challenge may also be the repeatability of conducted tests and testing a large number of samples, with the latter being particularly important in commercial laboratories, but not only.

**Figure 1:**
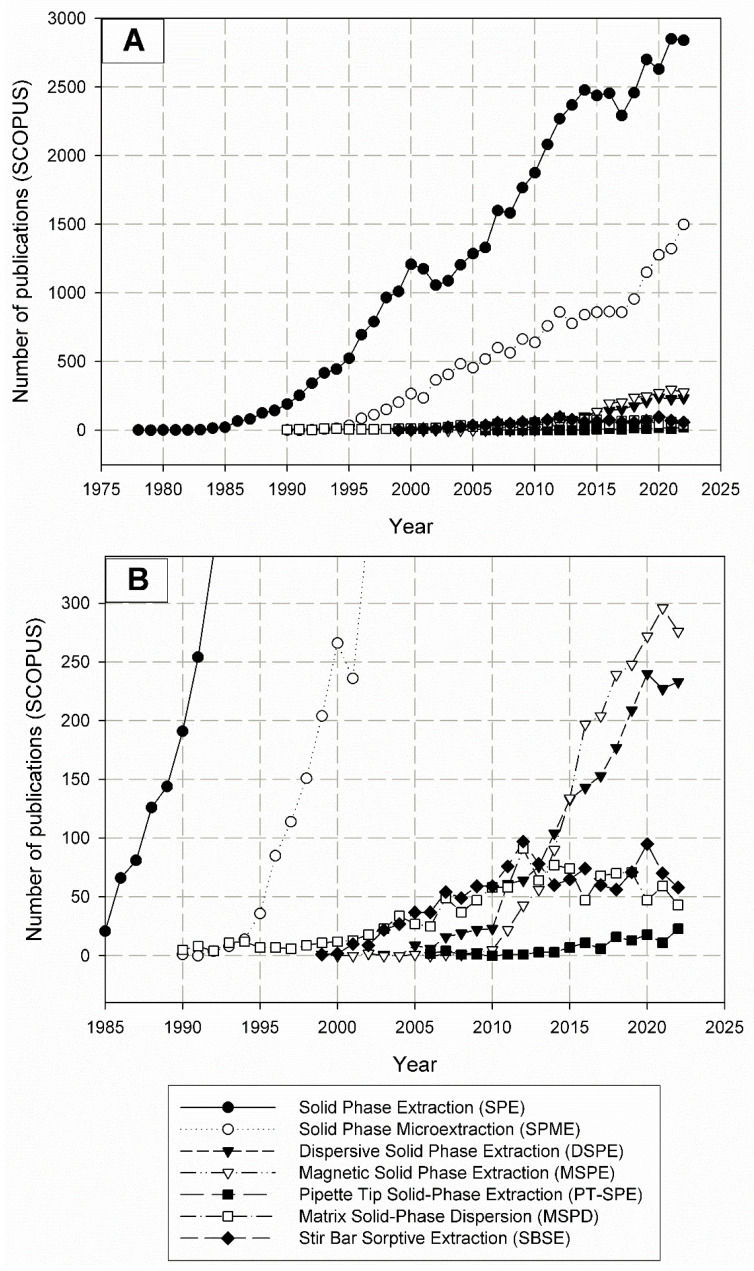
(**A**) Number of recently published scientific papers, including chosen techniques for preparing samples for analysis. (**B**) Excluding SPE and SPME techniques to better show the scale for other techniques. SCOPUS database [own elaboration].

Water-based sample preparation techniques used in laboratories can be broadly divided into those based on liquid-phase extraction and those based on solid-phase extraction [2]. In part thanks to the fact that there are a wide range of possible sorbents, solid-phase extraction techniques have been the main direction of development in recent years (Figure 1). As such, this article focuses exclusively on using variations of the solid-phase extraction technique.

Additionally, the miniaturization and adaptation of the properties of the sorption material, depending on the specific application—such as increasing the selectivity and specificity of the sorbent - have become the focus of researchers in recent years. The most common and most developed analytical techniques are variations and attempts to improve the SPE technique; these can include, for example, SPME, MSPE, PT-SPE, MSPD, and SBSE [2].

**Figure 2:**
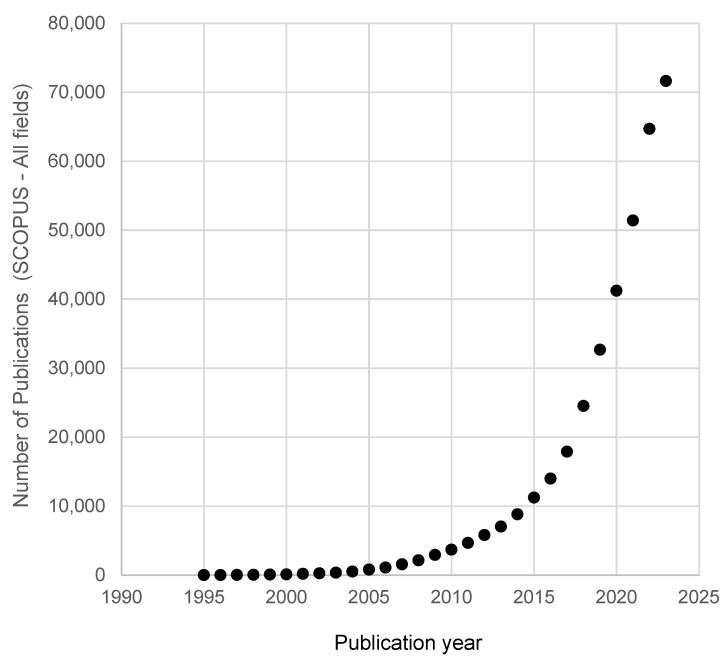
A number of scientific publications, including the term “Metal-Organic Framework,” since 1995. SCOPUS database, using “All fields” as the search field [own elaboration].

**Figure 3:**
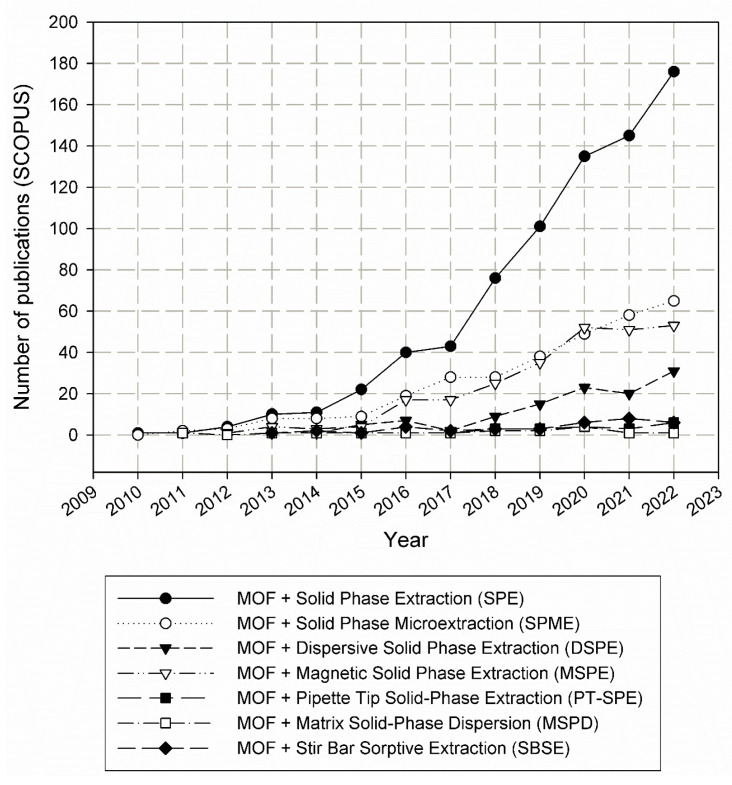
A graph showing the number of publications containing the keywords “Metal-Organic Framework” and the selected technique for preparing liquid samples for analysis. SCOPUS database, [own elaboration].

Various sorption materials are available commercially and have been adapted to many applications. However, work is still underway on both the form of sorbent application (miniaturization) and its properties (sorption efficiency, selectivity) [3,4,5]. Particularly interesting here seems to be the use of metal-organic frameworks (MOFs)—crystalline porous materials with high specific surfaces [6,7]. The popularity of the discussed sample preparation techniques and MOFs as sorption materials is very well illustrated by the graphs shown in Figure 1, Figure 2 and Figure 3.

MOFs, despite being a relatively young group of sorptive materials, have undoubtedly gained increasing interest in the scientific community (Figure 2), thanks to their remarkable properties compared to other sorptive materials and their potentially wide range of applications in many fields of science and technology [7,8]. The analysis of scientific publications on both MOFs and various sample preparation techniques allows us to observe that MOFs are eagerly used as sorptive materials in SPE technique (Figure 3). In second place, with a very similar number of publications, is the use of MOFs in SPME and MSPE techniques. The MSPE technique may not be as popular in combination with commercial sorbents due to the smaller amount of sorption material used in it, which may translate into a lower extraction efficiency. On the other hand, the binding of an MOF with another material may positively affect the durability of MOFs in the aquatic environment, and their high surface area could still provide a satisfactory extraction efficiency, even with fewer sorbents used.

Each of the sample preparation techniques has its advantages and disadvantages and is best suited for specific applications. Therefore, it is extremely important to understand all the techniques in detail and the possibilities of using different forms of sorbents in them in order to be able to make the most appropriate decision on how to conduct the analysis. Almost everywhere, one will come across a description of a study in which the authors explain that they were able to achieve analyte recoveries of ~100%, with low LODs and LOQs and high precision. In fact, one researcher’s developed methodology may be effective for the analysis of one compound or group of compounds but may prove to be completely inadequate for another. In addition, almost no studies mention the cost of the analysis being conducted, the availability and price of chemical reagents, the time-consuming nature of the tests being performed, or the complexity of preparing a particular extraction device.

This review article is intended to summarize and present the most commonly described uses of MOFs as sorbents in the most popular sample preparation techniques for analysis and to help the reader select the most appropriate technique and MOF for their particular applications. This review article is more comprehensive than the available review articles. It covers many water-based sample preparation techniques and does not focus on narrow areas of application of these techniques. The only limitation is that we describe the preparation of water-based samples for chromatographic analysis. Other review articles on MOFs and their applications as sorbents set out more detailed information on how to synthesize MOFs, modify them, and introduce defects into their structure that alter their properties [9], food applications [10], and environmental sample purification processes [11]. Additionally, significant information about MOFs can be found in a book by Yaghi et al. [12].

## 2. Metal-Organic Frameworks

The development of MOFs was initiated in the early 1990s. The founder of the MOF is considered to be Omar M. Yaghi, who, in 1995, together with his team, introduced the term “Metal-Organic Framework” for the first time, for the created complex compound of copper and 4,4-bipyridyl, which was then still showing unstable porosity [13]. The first permanently porous MOF was obtained in 1997; it was a complex compound of cobalt and bipyridyl. For the first time, it was possible to carry out the reversible adsorption of small gas molecules, CH_4_, O_2_, and N_2_ [14]. The beginning of extensive research on MOFs is considered to be Yaghi’s work, in which the MOF-5 compound was described, characterized by exceptional durability and high porosity at that time, where the specific area of the material was ~2900 m^2^/g [15]. Currently, the specific surfaces of some MOF materials can reach ~7000 m^2^/g (several times more than the specific surfaces of, for example, activated carbon) and a pore volume reaching up to 2 cm^3^/g [16]. The characteristic features of MOF are crystallinity, stable porosity, strong metal-organic ligand bonds, and a three-dimensional structure. Due to the number of possible combinations of metal clusters and organic ligands capable of forming MOFs and the possibility of modifying existing structures, it is theoretically possible to adjust their structure and properties depending on the need [17]. From this perspective, they are more interesting than, for example, zeolites. However, they are characterized by lower thermal stability than zeolites, and lower resistance to water [7]. An MOF consists of two principal parts: an ion or cluster of metal, a “node”, and a selected organic compound having at least two functional groups or two atoms capable of donating an electron pair, “connectors” or “ligands”. ‘Nodes’ and ‘ligands’ are connected by one or more coordination bonds. An MOF is characterized by a rigid crystalline structure with an ordered pore arrangement. The most common metal ions included in MOFs include Zr^4+^, F^3+^, Cr^3+^, Al^3+^, Cu^2+^, Zn^2+^, Ni^2+^, Mn^2+^, and Ag^+^. The type of element used, its valence, as well as the coordination number (which also depends on the ligand’s structure) affect the final shape of the MOF network. Metal ions forming MOFs very often do not occur alone but in the form of clusters. A “cluster” is a compound containing groupings of atoms of a given element connected directly to each other. They can be formed by elements from the p, d, and f blocks. Metallic clusters are chemical forms containing a certain number of metal atoms that, in a “significant percentage”, have an M-M (metal-to-metal) bond. M-L (metal-ligand) bonds can also be found in clusters. The basic feature of cluster compounds is a closed form, formed from M-M and M-L-M bonds [18]. In short, a metal cluster is a group of particles that form a shared network of connections, forming inert or charged molecules. Metals can combine in them, for example, with O, OH, or CO [19]. In relation to MOFs, they are often referred to as the “Secondary Building Unit” (SBU), whose spatial shape and properties can theoretically be selected to synthesize networks with specific properties [12]. An organic molecule, which is a ligand in the MOF, should contain at least two functional groups, or atoms, capable of donating an electron pair to form a coordination bond (e.g., N, O, OH, COOH). Aromatic compounds, one or more cyclic with at least two functional groups, are often used. An important parameter of ligands is their spatial structure, length, and the way of arrangement of their substituents. The use of the same SBU and similar organic ligands, e.g., terephthalic acid, which may contain different substituents, e.g. (-Br, -NH_2_, -COOH), but differing in length, leads to the formation of MOF families with the same lattice geometry but with different pore sizes. An example of such materials is isoreticular metal-organic frameworks (IRMOF) [7,20]. Despite the existence of potentially millions of different combinations of metallic nodes and organic linkers, due to their impermanence or difficulty in synthesis, only a small part of them has gained popularity and is available on the commercial market. One of the advantages of MOFs is the possibility of modifying their structure in a wide range, which leads to a change in their properties and potential applications. In the case of using MOFs as sorbents in the process of preparing liquid samples for analysis, changes in the sorption selectivity of selected analytes are obtained. Modifying the MOF structure to change network properties can be done in several ways. The simplest method of modifying the resulting crystal structures is the appropriate selection of substrates for their production, but an MOF can also be modified after the synthesis is completed, that is, through post-synthetic modification (PSM) [9].

Due to the rapid development of this group of compounds and the relatively short history of their existence, the problem remains with the naming and affiliation of MOFs compared to other porous materials. We are also dealing here with a situation in which the rapidly growing amount of research on MOFs has exceeded the number of attempts to systematize and classify their nomenclature. The situation probably needs to be simplified by the unusual structure of the compounds in question. While exploring this topic, there have been several attempts to link MOFs with other materials and to give them appropriate names, although these are still only guidelines. To date, there are no strict rules governing how an MOF is named. One can encounter a situation where one structure can be correctly described with several names [16,20,21,22]. The problem also concerned how to write the name of this group of compounds. Is the correct form a “Metal-Organic Framework”, a “Metal Organic Framework”, or an “Organometallic Framework? Eventually, it was determined that the correct form contained a hyphen in the middle: “Metal-Organic Framework”. The “Organometal” form has been reserved for complex compounds in which there is a direct M-C (metal-carbon) bond [16]. However, to this day, in some sources, both concepts are erroneously used interchangeably. The problem with the terminology of MOF also arose during the creation by individual universities and research institutions of common names and acronyms of materials developed by them, most often derived from the names of individual institutions, but not only. However, due to the high complexity of the structures of MOFs, acronyms formed from the names of individual universities and institutes have become an acceptable and popular way of naming this group of compounds [23].

The extraordinary properties of MOFs also translate into several practical applications. MOFs have found their applications mainly in gas storage (CO_2_, H_2_, CH_4_, and others), in gas separation and purification processes, as catalysts in organic reactions, as drug carriers, as sensors (luminescent properties) [12,24,25], and recently as sorption materials in chemical analysis [20,23]. In broadly understood chemical analysis, MOFs have proven themselves as sorbents during environmental contamination studies, food analysis, and in the chromatographic separation process. MOFs have also been used to remove environmental pollutants, in the process of wastewater treatment, the adsorption of hazardous materials from water and organic matrices, the removal of toxic and radioactive metal ions, aromatic compounds, air pollutants, and others [26]. For several years, publications have been made on the possibility of catalytic degradation and hydrolysis of chemical warfare agents (CWAs), using specially adapted MOFs [17].

*Durability of MOFs in the Aquatic Environment*

**Figure 4:**
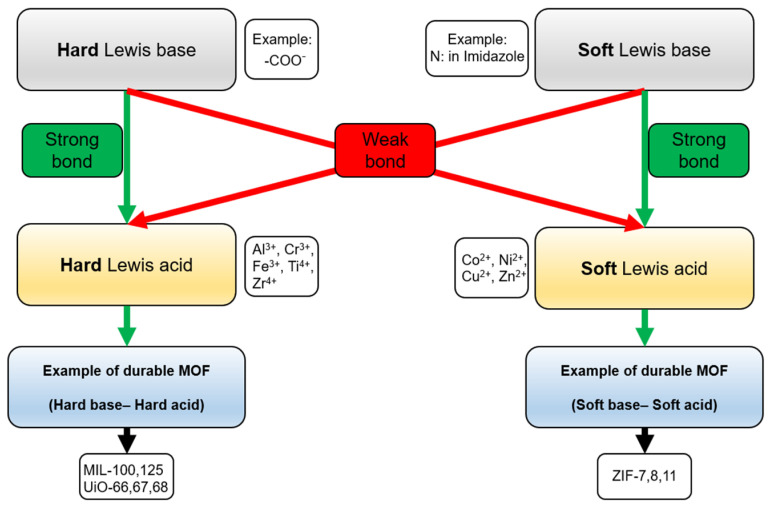
Schematic representation of MOF durability, according to the theory of HSAB, prepared based on the literature [28].

When using MOFs as sorbents in the preparation of water-based samples for analysis, their durability, especially their water resistance, is extremely important. This determines whether the selected MOF, firstly, can be used as a sorbent in the process of preparing water-based samples and, secondly, whether its structure will remain undamaged or will gradually degrade. And if it degrades, how will it affect the sorption properties, and how long can the selected MOF serve as a sorbent? Factors affecting the stability of the MOF in water are divided into thermodynamic and kinetic. In the case of thermodynamic agents, it is said to be the stability or instability of the MOF that matters. In the case of kinetic factors, it is the indifference or lability of the structure that counts. Thermodynamic factors include, for example, the pKa of an organic ligand, the oxidation state, and the atomic radius of the metal; kinetic factors include, for example, steric obstacle or hydrophobicity. However, it is possible to design an MOF to increase its resistance to water [12,27]. The stability of the MOF depends, among other things, on the strength of the coordination bond between the metal ion (or cluster) and the organic ligand. The strength of this bond depends on the charge density of the metal cation. The bond strength is proportional to the charge of the metal cation (its value) and inversely proportional to the radius of the ion. Metal ions with a higher valence (with a higher charge), e.g., Ti^4+^, Zr^4+^, Fe^3+^, Al^3+^, and Cr^3+^, form a coordination bond with organic ligands containing functional groups such as carboxyl group (hard base), and metal cations with lower valences (less charge), e.g., Ni^2+^, Cu^2+^, Mn^2+^, Zn^2+^, and Ag^+^, form coordination bonds, for example, with a free electron pair of nitrogen in imidazole (soft base), forming stable MOFs. This is consistent with Pearson’s theory of hard and soft acids and bases (HSAB). MOFs whose structure is consistent with this theory are characterized by higher mechanical, chemical, and thermal stability [28], making them suitable for use as sorbents in the preparation of liquid samples for analysis (Figure 4). In practice, one of the most important parameters proving the durability of the MOF is the strength of the coordination bond formed between the linker and the node. The strength of this bond can be controlled by increasing the pKa of the organic ligand, which is why ZIF materials show high durability for hydrolysis and thermal degradation in practice.

## 3. Application of MOFs as Sorbents in Selected Techniques of Preparing Liquid Samples for Analysis

### 3.1. Solid-Phase Extraction (SPE)

SPE is one of the most popular liquid sample preparation techniques for liquid-solid extraction. Sorption of analytes occurs due to intermolecular interactions between the analyte particles and the sorbent [3,29,30]. The SPE technique is extremely versatile due to the possibility of selecting the appropriate sorbent and elution solvent for the analytes to be tested. In general, usable sorbents (commercially available) can be divided into three categories: low-specificity sorbents, inorganic oxides, and specific sorbents. The simplest sorbents are inorganic oxides, such as silica gel and clay. They have a polar character and can be used during the sorption of polar analytes contained in non-polar solvents. Porous polymers and modified silica gels belong to sorbents with low specificity. Silica gel-based sorbents, functionalized by hydrocarbon chains, C_2_, C_8_, or C_18_, are particularly often used. They are used to extract non-polar analytes from water matrices, e.g., common organic water pollutants. The last group includes specific sorbents, which are ion-exchange resins. For this purpose, silica modified with organic groups containing, for example, NH_2_ or SO_3_H are used [31].

One of the main advantages of the SPE technique is its ability to concentrate the sample repeatedly. This technique also allows for a quick change of the sample matrix, characterized by high recoveries and repeatability. It is possible to automate the sample preparation process with SPE. For effective sample preparation using the SPE technique, a vacuum or overpressure should be applied to accelerate the flow of the sample through the sorbent bed. For this purpose, vacuum collectors are used, for example, which, combined with a vacuum pump, make it possible to collect the liquid phase from several columns simultaneously. In the SPE process, the amount of organic solvents needed is usually smaller compared to the popular LLE technique, and there is no risk of emulsion formation, which hinders the sample preparation process. Samples prepared with this technique can be analyzed with both GC and LC (depending on the extraction solvent used). The SPE technique is widely used in pharmaceutical, clinical, research, forensic, environmental, or food quality control laboratories in the preparation of samples for analysis and the determination of trace amounts of, among others: pharmaceuticals and metabolites, drugs, environmental contaminants (PAHs, pesticides, PCBs, phthalates), and vitamins [32].

Not coincidentally, SPE is the most popular. A significant factor in its selection is also that it is the easiest to apply. Placing the sorption material in an empty syringe between two sintered disks is relatively easy to implement in any analytical/research laboratory. However, this technique has one significant drawback. Its use of sorbents that exhibit a large specific surface area can lead to the “back pressure” phenomenon, i.e., column clogging. The vacuum or positive pressure created to force the sample through the column may not be sufficient. Using sorbents characterized by tiny pores or small grain diameters can lead to clogging when analyzing samples with a complex or heavily contaminated matrix. SPE requires the use of organic solvents in a few to several mL per sample. In most cases, it is also necessary to use a relatively large amount of sorbent; these values, depending on the type of sorbent and the size of the column, can range from several dozen milligrams to a couple of grams.

The use of MOF in the SPE technique may be limited simply to placing the sorbent in the SPE column, between two porous disks; it is optional, for example, to create magnetic composite materials as in the case of the MSPE technique. MOFs are materials characterized by the presence of a complex spatial structure of micro and macro pores, which can negatively impact the rate at which the water sample will flow through the column (permeability, or the resistance that the sorbent puts to the flowing sample). However, the use of vacuum collectors can solve this problem to some extent. One of the disadvantages of using MOF in the SPE technique is the relatively large amount of sorption material that needs to be placed inside the column. In the standard case, the amount of sorbent should be between a few tens of milligrams to several grams, which is why there has been a trend towards the miniaturization of sample preparation systems in recent years. The high specific surface area of MOFs, combined with the significant concentration capacity of SPE, is an excellent combination for the analysis of compounds present in samples at very low concentration levels.

**Figure 5:**
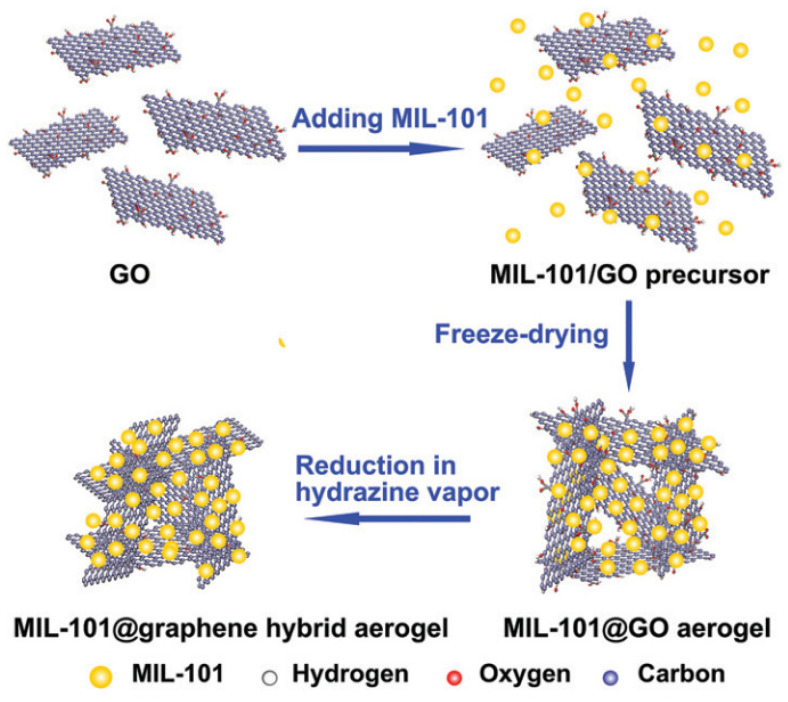
Synthetic procedure for MIL-101@GO presented by Zhang et al. [33].

MOFs, in combination with the SPE technique, are mainly used for the determination of compounds in aqueous samples [33,34,35,36,37,38]. Materials from Institut Lavoisier (MIL) [33,34,37,39] and from the University of Oslo–UiO [38,40,41] are widely used. MIL-101(Cr) was used in tandem with graphene hybrid aerogel to determine selected non-steroidal anti-inflammatory drugs (NSAIDs) and selective enrichment/concentration of selected proteins (ribonuclease A enrichment while excluding cytochrome C and lysozyme) [33]. A graphene aerogel (GA)-supported MOF, demonstrating a 3D structure, was made using a simple ‘sol-cryo’ technique, which does not require the use of “guest” molecules whose role is to direct the growth of MOF crystals (Figure 5). Thanks to the use of the described sorbent, and using the HPLC-UV-Vis technique, the authors of the study managed to obtain satisfactory recoveries (89.2–100.7%), high sensitivity of the method, and good repeatability (3.7–8.5%) for five selected NSAIDs.

**Figure 6:**
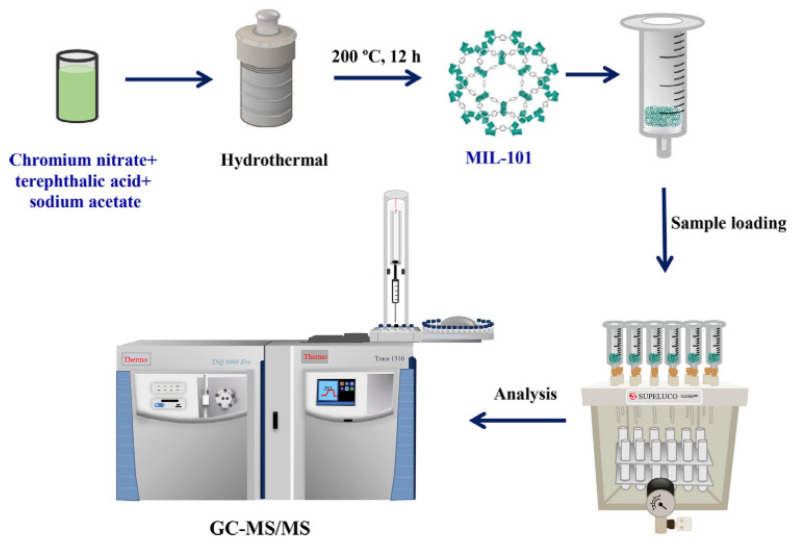
A simple MIL-101(Cr) synthesis and application protocol presented by Nurerk et al. [37].

Another promising application example of MIL-101(Cr) is the analysis of sulfonamides [34]. The authors used both MIL-101(Cr) and the related MIL-101(Fe) to determine four selected sulfonamides in environmental water samples using the UPLC-MS/MS technique. Using the described MOF, the authors were able to determine sulfadiazine (SDA), sulfamethazine (SMZ), sulfachloropyridazine (SCP), and sulfamethoxazole (SMX), with a LOD of ~0.03–0.08 g/L. The developed analytical method was characterized by recovery for the examined analytes at the level of 83.5 to 107.3%, with repeatability expressed in RSD at the level of 0.2 to 8.0%. An extremely interesting aspect of this study was the application of computer modeling to predict the affinity of MIL-101(Cr) for selected sulfonamides, which not only accurately predicted the studied relationships but also provided a new strategy in the analysis of compounds at very low concentration levels. MIL-101 has also found its application in the determination of eleven selected UV-filtering compounds, also from aqueous samples [37]. This MOF was placed directly in a polystyrene SPE column and put down in a vacuum collector [37]. The eluted compounds were determined using GC-MS/MS. The dependence of several factors on the extraction process was investigated, such as the desorption conditions of compounds, the pH of samples, salting, and the volume of the tested samples. The LOD determined during validation ranged from 1.0 to 11.7 ng/L, and the analytes were determined with a satisfactory recovery of 82 to 105%. The highest concentration of the tested compounds was detected in water samples from swimming pools. The method of simple MIL-101(Cr) synthesis and analytical workflow for the developed SPE-GC-MS/MS method is shown in Figure 6. 

**Figure 7:**
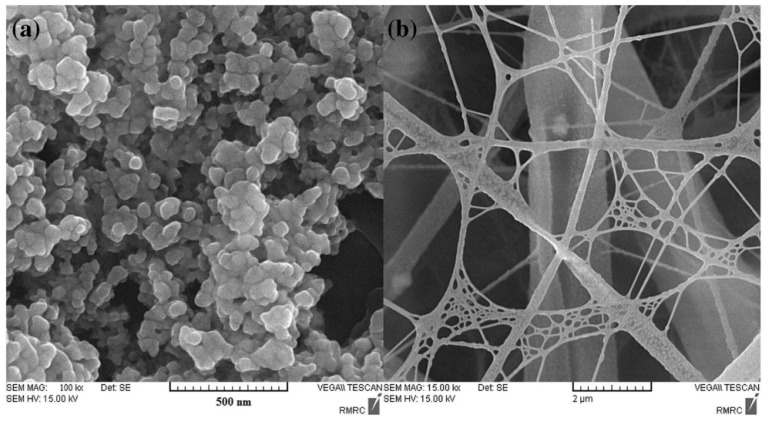
SEM images of synthesized MIL-68 (Al) nanoparticles (**a**) electrospun spider-web-like chitosan/MIL-68 (Al) composite nanofibers (**b**) [39].

The synthesis of spider-weblike chitosan/MIL-68(Al) composite nanofibers and their application to fast SPE was described by Asiabi et al. [39]. The authors of the paper describe the possibility of using MIL-68(Al) in combination with chitosan, creating a composite material in the form of nanofibers suitable for use in the SPE technique for determining the content of Pb^2+^ and Cd^2+^ in samples of Trochus erythreus, a selected species of sea snail. The tested compounds were determined using the ICP-OES technique; the LOD was 0.16 μg/L for both compounds. The authors used the electrospinning technique to produce their composite material, thanks to which the resulting material resembles a spider’s web with a developed surface, which significantly promotes the process of sorption of compounds on the material used (Figure 7).

UiO-66 has also found its applications. It is often synthesized in several versions, differing from each other by the presence of one group -NH_2_ or -OH connected to the aromatic ring in the ligand of this MOF [38,40,41]. UiO-66-OH has found its application in the extraction of PFCs and PFOS from aqueous samples. UiO-66-OH was used in conjunction with the MALDI-TOF-MS technique to determine macromolecules, including the described PFCs [40]. This material has been successfully used both as an adsorbent and as a matrix for the MALDI-TOF-MS technique. The research found that MOF shows high laser desorption/ionization efficiency and functions well in both positive and negative ion modes. Compared to MS spectra with conventional matrices, MS spectra obtained on the MOF matrix are characterized only by deprotonated molecule ion peaks, without background interference. UiO-66-NH_2_, combined with molecularly imprinted polymer (MIP), was used as a sorbent to extract four selected aflatoxins (AFB1, AFB2, AFG1, and AFG2) from grain samples [41]. As a result of the combination of MIP and MOF, a polymer with surface printing type UiO-66-NH_2_@MIP was obtained. Quercetin was used as a virtual template, and the MOF acts here as a carrier into which the acrylamide monomer can be copolymerized. The sorbent was successfully used to determine the discussed analytes. The developed analytical method was characterized by good linearity in the range of 0.20 to 45 μg/kg. LOD ranged from 90 to 130 ng/kg. The obtained recoveries ranged from 74.3 to 98.6%. UiO-66 and UiO-66-NH_2_ were also used in discs with different mixed-matrix discs (MMD) to perform automated flow-through extraction of 7 selected substituted phenols in aqueous samples. UiO-66 crystals of various sizes (90, 200, and 300 nm) have been embedded in mechanical polyvinylidene discs (PVDF). MOF-MMDs enabled the simultaneous extraction of phenols while excluding larger molecules. The best results were obtained with 90 nm UiO-66-NH_2_ crystals. Using the obtained sorbent, LOD was obtained in the range from 0.1 to 0.2 μg/L. Relative standard deviations of the method ranged from 3.9 to 5.3% intra-day, and 3.7–5.7% inter-day. The obtained recoveries for the studied analytes ranged from 90 to 98%. 

A summary of the MOF materials used in SPE is presented in Table 1.

### 3.2. Solid-Phase Microextraction (SPME)

The technique of SPME was developed by the team of Prof. Pawliszyn in the early 1990s [49]. The SPME technique itself is based on the phenomenon of the sorption of analytes on the surface of a thin fiber. This process can be described as the equilibrium constant or the split constant of the analyte, depending on how the technique is used, between two or three phases in a direct immersion (DI) or headspace (HS) system. The HS allows the absorption of analytes found in lubricants, thick oils, and soil but limits the use of SPME to volatile and medium-volatile compounds. The great advantage of SPME is also its ability to concentrate analytes. Commercially available fibers for SPME allow multiple concentrations of analytes present in the headspace. In addition, the use of this technique is extremely simple, fast, and does not require the use of any organic solvents. Fibers with adsorbed analytes can be directly introduced into the GC inlet for the analyte desorption. Thanks to its advantages, SPME remains one of the most popular techniques for determining volatile and medium-volatile compounds. This technique constantly evolves, especially towards developing new sorption materials [50,51]. Most publications on applying the SPME technique are the result of analyses of volatile and medium-volatile organic compounds (e.g., BETX) in combination with gas chromatography. Most MOFs, in addition to their large specific surface area and the possibility of their modification, are also characterized by relative instability in the aquatic environment. The current applications of MOF focus mainly on the sorption and separation of gases [12]. Combining the HS-SPME technique with the use of MOF as a sorption layer seems to be exceptionally highly beneficial due to the possibility of the sorption of volatile and medium-volatile compounds from the headspace, and consequently not exposing the MOFs to direct contact with the aqueous matrix of the samples. Sorbents used in the SPME technique must have high thermal stability (in the range of 250–350 °C) because the desorption of analytes most often occurs thermally in a gas chromatograph inlet, and MOFs demonstrate durability at the required temperatures.

When working on using new sorbents in the SPME technique, it is also important to consider how to apply the sorbent to the fiber. Often, difficulties in applying a sorbent to fiber can be a significant obstacle to the development of this technique. There are several different approaches to this problem. One of the most straightforward solutions is to cover the fiber with silicone or epoxy glue (it is essential that the adhesive used be resistant to high temperatures in the range of ~250–350 °C), then immerse the fiber coated with glue, e.g., in the powder of the MOF. This process can be repeated several times to obtain the desired thickness of the sorption layer [52]. Another approach involves immersing the metal fiber in a polymer and MOF composite solution [53]. Direct solvothermal synthesis of the MOF on the fiber’s surface is also used, followed by direct carbonization of the sorbent layer formed in this way on the fiber [54]. The creation of composite materials made of MOF, such as graphene oxide (GO), also gives great hope. Graphene oxide is chemically bound to silica present on the surface of the fiber and to the selected MOF [55].

**Figure 8:**
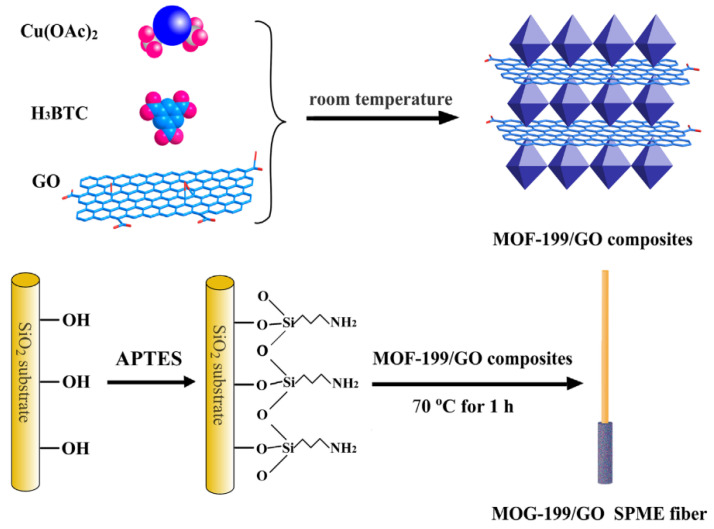
Schematic representation of MOF-199/GO composite synthesis for SPME extraction using APTES as a linking agent, presented by Zhang et al. [56].

Due to the specificity of the SPME technique, it is most often used in combination with gas chromatography. The use of MOFs in SPME is successfully used not only for testing water samples but also fruit [56], plasma and urine [57], milk [58], soy oil [59], honey [60], and fish [52]. The most frequently studied analytes include polycyclic aromatic hydrocarbons [52], organochlorine pesticides [56], fragrances [54,61], polybrominated diphenyl esters [58], and fluoroquinolones (FQs) [60]. In the SPME technique, the shape and dimensions of the fiber itself force researchers to develop a method of permanent connection of the MOF with the surface of the fiber. An example is the combination of MOF-199 with graphene oxide used in the study presented in Figure 8 [56]. The authors used 3-amino-propyl-triethoxysilane (APTES) as a cross-linking agent, which increased the durability and allowed the fiber to be reused more than 140 times. Composite materials created from MOFs and GO showed an increased affinity for organochlorine pesticides, resulting from the combination of the high specific surface area of the MOFs and the unique nature of graphene oxide. The developed analytical method was characterized by LOD at the level of 2.3 to 6.9 ng/L, and the relative standard deviation for five extractions carried out using the same fiber ranged from 5.3 to 8.8%. Recoveries for the analytes studied ranged from 72.2 to 107.7%.

**Figure 9:**
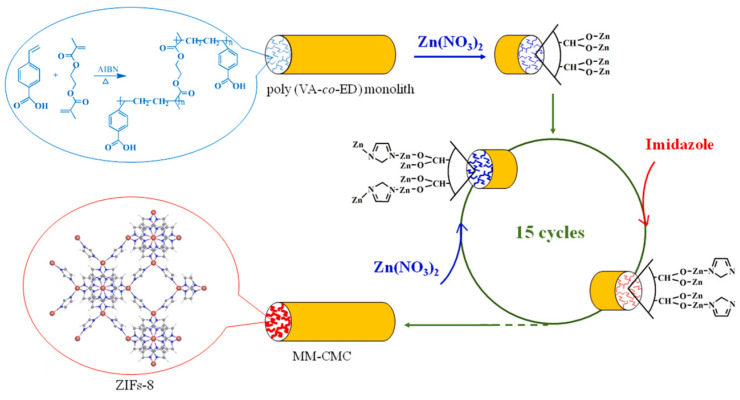
The preparation sketch of MOF-monolith composite-based capillary microextraction column (MM-CMC) by Pang et al. [60].

The combination of graphene oxide with MOF, more specifically with MIL-88(Fe), was also used in another study [55]. The authors managed to connect the MIL-88(Fe)@GO composite with the steel fiber SPME using a covalent bond. The steel SPME fiber was first coated with a layer of silver, then coated with a layer of silica using mercaptopropyltriethoxysilane (MPTES) and APTES. Then, the fiber prepared in this way was immersed in MIL-88(Fe)/GO and heated to 100 °C for 1 hour. The fiber obtained in this way has been successfully used to determine phthalic acid esters (PAE) from various vegetable oil samples. The developed analytical method was characterized by LOD at the level of 0.5–2.0 ng/g, repeatability in the range of 4.0 to 11.4%, and analytes were determined with recovery at the level of 83.1 to 104.1%. Another approach to creating composite materials containing MOF for use in SPME is presented in an interesting article published by Pang et al. [60]. In this study, the authors determined the content of fluoroquinolones in water and honey samples using a metal-organic composite in the form of a monolith for microextraction in a solid-phase tube (IT-SPME). For this purpose, 4-vinylbenzoic acid was copolymerized with ethyl methacrylate in a molten silica capillary to form a porous monolith. Subsequently, the in-situ ZIF-8 pore surface of the monolith was synthesized (see Figure 9). The introduction of ZIF-8 increased the composite surface area. The developed analytical method allowed for the detection of compounds with LOD at the level of 0.14 to 1.1 ng/L. Relative standard deviations for all the samples were less than 10%, with recovery ranging from 80.1 to 119%.

**Figure 10:**
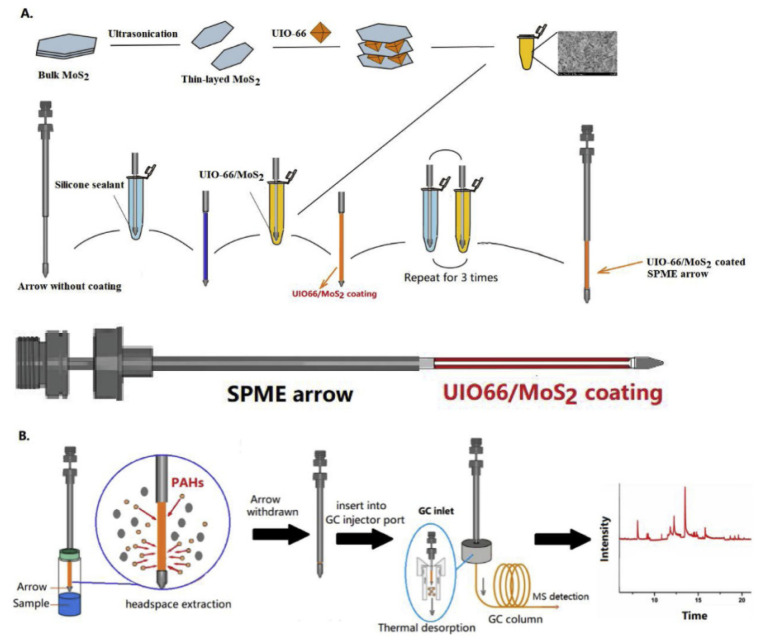
A SPME arrow-like system presented by Yuan et al. [52].

UiO-66-OH has also found its application in SPME. The authors used this MOF to determine polybrominated diphenyl ethers (PBDEs) from milk [58]. In this case, it was not decided to produce a composite material but to combine UiO-66-OH with the surface of the steel SPME fiber using silicone glue. Under optimized conditions, the developed method was characterized by excellent linearity R^2^ = 0.9994, LOD in the range of 0.15 to 0.35 ng/L, and recovery at the level of 74.7–118.0%. The developed method is reliable and effective for determining PBDE in milk at very low concentration levels. The UiO-66 was also used in another study (Figure 10) [52]. In this case, it was used in the SPME-Arrow technique, a variant of SPME characterized by a different shape of sorption fiber. MOF was used to form a composite and combined with molybdenum disulfide MoS. This technique, combined with gas chromatography, allowed for the determination of the content of sixteen selected aromatic hydrocarbons in fish samples. The composite material was bonded to the surface of the SPME fiber using silica gels. The manufactured SPME sorption fibers showed an increased specific surface area (from 2.1 to 4.5 times) compared to commercial fibers with PDMS/CAR/DVB coatings. Selected compounds were detected with LOD ranging from 0.11 to 1.40 ng/kg, with a relative standard deviation of <8.6% and recovery ranging from 80.2 to 101.0%.

Some MOFs, despite their high specific surface area, are also characterized by low water resistance. One of the ways to maintain a high specific surface area of the sorbent and increase its resistance to hydrolysis is the carbonization of MOF [54]. The authors first in-situ synthesized MOF-74 on the surface of the SPME fiber, then subjected it to the carbonization process. The obtained sorption fiber MOF-74-C was characterized by good thermal and chemical stability. It has been successfully used to analyze selected fragrance compounds. The developed method was characterized by a wide linearity range (more than two orders of magnitude), a relative standard deviation below 8.7%, with recovery ranging from 83.6 to 115.5%. In another study, the rare CIM-80(Al) was used as a sorbent in the HS-SPME analysis of selected six methylsiloxanes and seven fragrances in aqueous samples. In combination with the GC-MS technique, the MOF coating used was particularly effective in the determination of volatile methylsiloxanes, showing moderately lower LOD (0.2–0.5 μg/L) and slightly better precision (RSD < 17%) compared to commercial DVB/PDMS fiber, with LOD = 0.6 μg/L and RDS value < 22%. CIM-80(Al) was applied to the surface of a fiber made of nitinol using a cross-linking agent, APTES. Initially, a silica layer was applied to the surface of nitinol using APTES; then, on the surface of the fiber prepared in this way, the MOF was synthesized in-situ.

A summary of MOF materials used in SPME is presented in Table 2.

### 3.3. Dispersive Solid-Phase Extraction (D-SPE) and Micro–Solid-Phase Extraction (μ-SPE)

In recent years, there has been a trend towards miniaturization and simplification of sample preparation techniques for analysis. These efforts are carried out, for example, to reduce the number of organic solvents used in sample preparation and to create the possibility of testing smaller and smaller quantities of samples with complex matrices. Not without significance, there is also a possibility of using smaller amounts of solid sorbents, which is particularly important if the cost of purchasing the sorbent is relatively high or the sorbent itself is somewhat challenging to synthesize, as in the case of MOFs. One of the results of such miniaturization is the μ-SPE technique [66,67].

The μ-SPE technique was initially introduced in 2006 as an alternative to multi-stage SPE [68]. Due to the development of microextraction strategies, and the large variety of ways of using them in practice, many types or variations of these techniques can be found [66]. Dispersive-Micro-Solid-Phase Extraction (D-μ-SPE) and porous membrane-protected Micro-Solid Phase Extraction have recently gained popularity. The second version does not have its acronym; it is often simply described as “porous membrane-protected”. D-μ-SPE is a miniaturized version of Dispersive-Solid-Phase Extraction (D-SPE). It uses a small amount of solid sorbent placed directly in a solution of an aqueous sample. Due to the direct placement of the sorbent in the solution, it is possible to obtain significant extraction yields to the solid phase. However, in this release, the sorbent is directly exposed to the matrix, and interferents are present in the sample. The sorbent is then filtered out, and the analytes are desorbed with an adequately selected solvent. μ-SPE protected by a porous membrane (usually polypropylene), consists of placing a small amount of sorbent (even a few milligrams) in a self-made “envelope” made of porous semi-permeable material (e.g., polypropylene). This technique is a simplified and miniaturized version of the SPE technique. It is simplified because the process of concentration, extraction, and purification from interferents (clean-up) takes place here in one step. A sheet of the porous membrane is properly folded, cut, and hot-welded to form a small envelope inside which the sorbent can be placed. The use of a plastic permeable membrane provides mechanical protection for the sorbent. Thanks to its use, it is possible to insert a container with sorbent directly into even very contaminated or complex samples, e.g., environmental or biological. In both D-μ-SPE and μ-SPE versions with a semi-permeable membrane, the sorbent can be mixed with the sample (to increase the sorption efficiency), of course using a mechanical stirrer, sometimes described as (vortex assisted, VA), but also by ultrasound (sonification assisted, SA), and microwave-assisted (MA) [66]. When using a semi-permeable membrane, the sorbent container is removed from the sample solution, and the analytes are desorbed using an organic solvent [67]. An additional advantage of this method is the possibility of using small amounts of sorbent (a dozen or several dozen mg), which favors the use of less common sorbents, the use of which in the SPE technique could be disadvantageous due to, for example, the cost of their purchase. The μ-SPE technique can include carbon sorbents such as graphene, graphite oxide (GO), carbon nanotubes (CNTs), including single-walled carbon nanotubes (SWCNT), and multi-walled carbon nanotubes (MWCNT), molecular mapping polymers (molecularly imprinted polymers, MIP), biopolymers and, which have become increasingly popular in recent years, MOFs [66,67].

**Figure 11:**
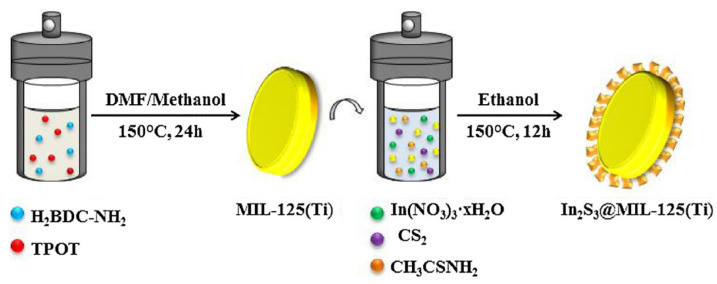
Synthetic protocol developed by Jia et al. for the core-shell In_2_S_3_@MOF nanocomposite [69].

Analyzing articles on the use of MOF in dispersion techniques for the preparation of liquid samples for chromatographic analysis, it is easy to observe the considerable popularity of MOFs, hiding under the acronym MIL—Materials from Institut Lavoisier, for example: MIL-125(Ti) [69], MIL-101(Cr) [70], MIL-100(Fe) [71], and MIL-53(Al) [72]. The second most commonly used MOF is UiO-66 [73] and UiO-67 [74]. Authors use those not only for water samples but also food products like meat [74], honey [71], oils [70], for synthetic urine [75], and human plasma [76]. The most commonly determined analytes are non-steroidal anti-inflammatory drugs [77], steroid hormones [78], mercury [76], herbicides [79], and progesterone [75]. The authors of this work [69] took an interesting approach to using MOF in the D-SPE technique. The authors used composite material as a disc, made by combining MIL-125(Ti) and In_2_S_3_ (Figure 11). The compound with indium was used to enhance the adsorption efficiency of aromatic analytes from water samples. The solvothermal method carried out both the synthesis of MIL-125(Ti) and binding it to the In_2_S_3_. The material thus developed was used to determine sixteen selected nitro-polycyclic aromatic hydrocarbons in aqueous samples. The developed analytical method, combined with the GC-NCI-MS/MS technique, allowed the detection of the tested compounds with LOD at the level of 2.9 to 83.0 ng/L, with RSDs <10% and recoveries for individual analytes, ranging from 71.3 to 112.2%.

**Figure 12:**
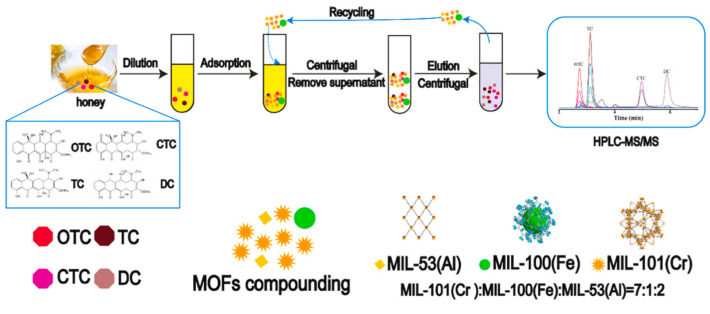
d-SPE procedure for the extraction of selected TCs (tetracyclines) from honey samples using MOFs as sorbents [71].

To determine selected tetracyclines in honey samples using the D-SPE technique, can be used a mixture of three MOFs: MIL-101(Cr): MIL-100(Fe): MIL-53(Al) in a ratio of 7:1:2 (Figure 12) [71]. The MOF mixture was placed directly in diluted honey samples, the samples were subjected to shaking and ultrasound. The procedure combined with HPLC-MS/MS, allowed obtaining a limit of detection in the range of 0.073-0.435 ng/g, for up to four types of honey samples, with recoveries for analytes ranging from 88.1 to 126.2%.

Progesterone is one of the hormones that plays an important role during pregnancy in mammals and in the growth and development of animals. Conventional analytical methods for the detection of this compound include GC-MS and HPLC-MS. The authors of this paper [75] presented an easy-to-perform procedure for determining progesterone in aqueous and synthetic urine samples using the D-μ-SPE technique using MIL-101(Cr)-NH_2_ as sorption material. Under optimized conditions, a wide linear range was obtained, from 0.5-500 ng/mL, with a relative standard deviation ranging from 2.4 to 8.4%. The recovery of the analyte ranged from 92.0 to 117.8% at three concentration levels (5, 25, and 100 ng/mL). These studies demonstrated an easy-to-apply approach to hormone analysis by combining the advantages of mass spectrometry and MOFs. In one of theworks [77], authors created a composite material—a cryogel for the first time by combining the MOF series with poly(vinyl alcohol, PVA). The MOF used are MIL-101(Cr), MIL-100(Fe), ZIF-8, MOF-199 and MIL-53(Al). The manufactured composite materials were combined with HPLC-MS/MS to detect four non-steroidal anti-inflammatory drugs in environmental water samples. The sorption properties of MIL-101(Cr)/PVA cryogel exceeded those of MIL-101(Cr) alone. Under optimal conditions, analytes were determined with LOD at the level of 0.007 to 0.037 μg/L, and recoveries of the tested analytes ranged from 78.44 to 105.70% with a relative standard deviation ranging from 1.33 to 9.85%. The possibility of using MOFs as sorbents was investigated to determine the content of four selected androgens and progestogens in aqueous samples. MIL-101(Cr), MIL-100(Fe), MIL-53(Al.) and UiO-66 were used. In further research, the use of UiO-66 was used. The MOF was placed in a “device”—a folded envelope made of thin polypropylene. In combination with the LC-MS/MS technique, under optimized conditions, it was possible to detect the tested analytes in aqueous samples in the linearity range from 7.624 to 2032 ng/L for selected compounds, in LOD in the range from 2.0 to 10.0 ng/L, RSD < 10.0% and recoveries in the test compounds in the range from 80.5 to 102.4%. The extraction yield using the μ-SPE UiO-66 “device” was compared with several classic SPE columns. In the case of selected analytes, the use of μ-SPE allowed us to obtain higher extraction efficiency. UiO-67 has found its application as a sorbent in the D-SPE technique for the analysis of sulfonamides in meat samples [74]. Thanks to the synergistic interaction of π-π, hydrogen bonding, hydrophobic interaction, and matching the size of sulfonamides to the pore size of stable and mesoporous UiO-66, satisfactory extraction performance was achieved. In combination with the HPLC-DAD technique, a simple method was obtained to determine these compounds in meat samples. The tested compounds were determined in the linearity range from 14.6 to 250 ng/g, with LOD in the range from 0.7-6.5 ng/g, RSD < 3.4%, and the recovery at the level from 83.4 to 103.4%.

A summary of MOF materials used in D-SPE and μ-SPE is presented in Table 3.

### 3.4. Magnetic Solid-Phase Extraction (MSPE)

**Figure 13:**
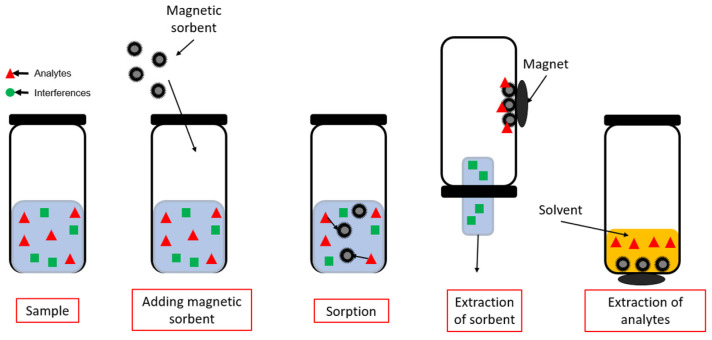
Application of the MSPE technique—variant [own elaboration].

In the D-SPE technique, solid sorbent grains are placed directly in the tested sample, where the sorption process of the tested compounds takes place. This technique ensures high extraction efficiency due to the large contact surface of the sorbent with the sample. This technique also has some drawbacks; the sorbent is most exposed to potential impurities present in the sample. The sorbent must then be filtered, which may vary according to the matrix’s composition and the test sample’s characteristics. A modification of this technique aimed at eliminating some of the disadvantages of D-SPE is MSPE. This technique is analogous to the D-SPE technique, with the difference that in MSPE, the sorbent exhibits magnetic properties, which allows it to be more easily separated from the sample solution, e.g., using an external magnet. MSPE imposes certain restrictions on the sorbent itself—it must exhibit magnetic properties (Figure 13). Sorbents showing magnetic properties can be used, or existing sorbents can be modified or built on, e.g., Fe_3_O_4_ nanoparticles [91]. The use of nano- or micro-particles, Fe_3_O_4_ (mixed nanoparticles, diameter~5–100 nm) of iron (II) oxide and iron oxide (III), to give the sorbent magnetic properties, has also become popular in the context of the use of MOFs as a sorption material, creating the Magnetic Metal-Organic Framework (MMOF). There are several ways to modify classical sorbents or MOF with Fe_3_O_4_ to exhibit magnetic properties [92,93]. Simple mixing of the initial sorbent with nanoparticles is the simplest method, although the resulting sorbent is the least stable. Often, Fe_3_O_4_ nanoparticles are coated with a silica layer, which acts as a “linker” that connects the magnetic Fe_3_O_4_ particles and the sorbent itself [91].

**Figure 14:**
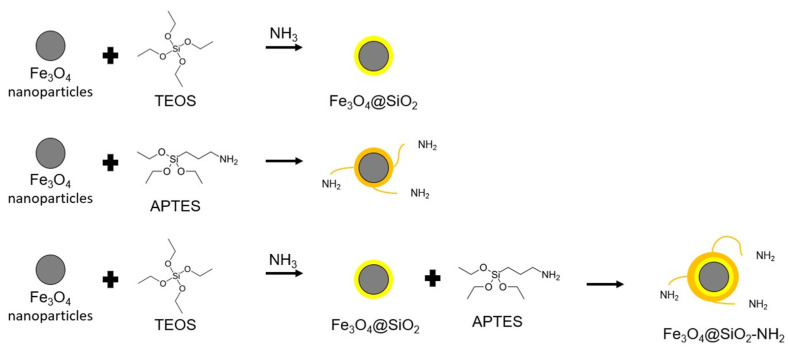
Application of a silica layer to magnetic nanoparticles [94,95,96].

Iron oxide nanoparticles are coated with silica in the Stober process [94]. Iron particles can either be directly coated with silica using tetraethoxysilane (TEOS) or APTES, resulting in NH_2_ group left outside the nanoparticle surface or first with TEOS, then APTES, leading to the formation of more regular, larger diameter nanoparticles with free NH_2_ [94]. Both methods of coating iron oxide nanoparticles with silica are shown schematically in Figure 14. NH_2_ groups can potentially be chemically bound to selected functional groups on the MOF surface to increase the bond stability of magnetic particles and metal-organic sorbent [12,56]. The process of applying another silica layer using APTES is analogous to TEOS, except that no ammonia is necessary to functionalize the nanoparticles using APTES. 1 mL ethanol and 4% APTES solution were added to 1 mL of aqueous 60 mg/L solution Fe_3_O_4_@SiO_2_. The temperature and reaction time were the same as before [95]. One paper briefly describes depositing a silica layer on iron nanoparticles in the Stober process: nanoparticles can be placed in a 200 mL aqueous solution of 0.1 M HCl to develop their surface; the nanoparticles are then drained and placed in water [96]. An aqueous solution of 60 mg/L of magnetic nanoparticles is placed in a round-bottomed flask, heated to 40 °C, diluted with 200 mL ethanol, and ultrasonicated for 15 min to disperse in a solid particle solution. In this order, 9 mL of 28% aqueous solution of NH_3_ and 1.2 mL of TEOS are added to the flask. The suspension in the flask was stirred and maintained at 40 °C for 2 h. The resulting Fe_3_O_4_@SiO_2_ precipitate is collected using an external magnet to avoid collecting silica particles that do not contain magnetic nanoparticles inside. The nanoparticles thus collected were washed three times with ethanol and water [96]. 

Fe_3_O_4_ can also be combined with MOF in the process of their solvothermal synthesis. Then, in addition to organic links and salts of the corresponding metals, magnetic nanoparticles are added to the reaction mixture, which is enclosed inside the formed crystals of metal-organic lattices during the precipitation of the MOF [93].

Increasing the durability of MMOF can be achieved by converting them into Magnetic Porous Carbon, MPC. MPC can be produced by carbonizing MOF or MMOF. As a result of carbonization, organic components (linker) are transformed into a porous carbon structure, enclosing in themselves metallic nano- or micro-particles with magnetic properties. In addition, metals present in clusters are leached. In the treasured MOF, hydrolysable coordination bonds are no longer present, and the porous structure is formed only with organic linkers. The obtained MPCs are characterized by more excellent durability, but unfortunately, they lose some porosity [93].

**Figure 15:**
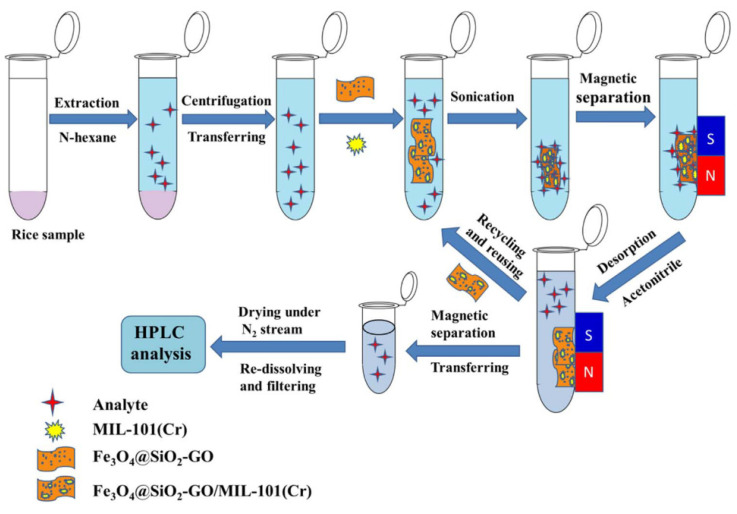
A simple extraction procedure showing a quick combination of MOF and magnetic particles, presented by Liang et al. [99].

Each time the use of the MSPE technique is associated with a magnetic field-prone component with ferromagnetic properties combined with the sorbent used. In each of the techniques discussed in this article, the role of Fe_3_O_4_ nanoparticles is discussed, and more information can be found at the beginning of this section. However, interestingly, two main ways of combining the sorbent (MOF) with Fe_3_O_4_ can be observed. The first of these is the use of silylating agents, such as TEOS and APTES, for binding the sorbent to the surface of iron oxide nanoparticles using a silica layer [97,98,99,100]. The second of which is the direct MOF precipitation in the presence of Fe_3_O_4_, thanks to which ferromagnetic nanoparticles are “trapped” inside the sorbent [97,101,102]. The frequently chosen MOF are MIL-101(Cr) [99], MIL-125-NH_2_ [103], and MIL-100(Fe) [102]. You can also meet with the use of ZIF-8 [97,100] and less common MOF-5 [101] and MOF-808 [36]. It is still popular to create composite materials, using e.g. graphene oxide [99,100]. The MSPE technique combined with MOF is used to detect organophosphorus pesticides [104], benzoylurea insecticides [36], triazole fungicides [100], and others, including NSAIDs [102] in aqueous samples [98], tea [97], rice [99], honey [100] and juice [36]. A practical example of the use of MOF is the composite material Fe_3_O_4_@SiO_2_-GO/MIL-101(Cr), created by combining the sorption properties of the MOFs and graphene oxide with the magnetic properties of iron oxide nanoparticles for the determination of selected seven triazine herbicides in rice samples (Figure 15). In combination with the HPLC-UV-Vis technique, LOD was obtained for the tested compounds at the level of 0.010-0.080 μg/kg, with the recovery of analytes in the range of 83.9-103.5%, with relative standard deviations of less than 8.7% [99].

**Figure 16:**
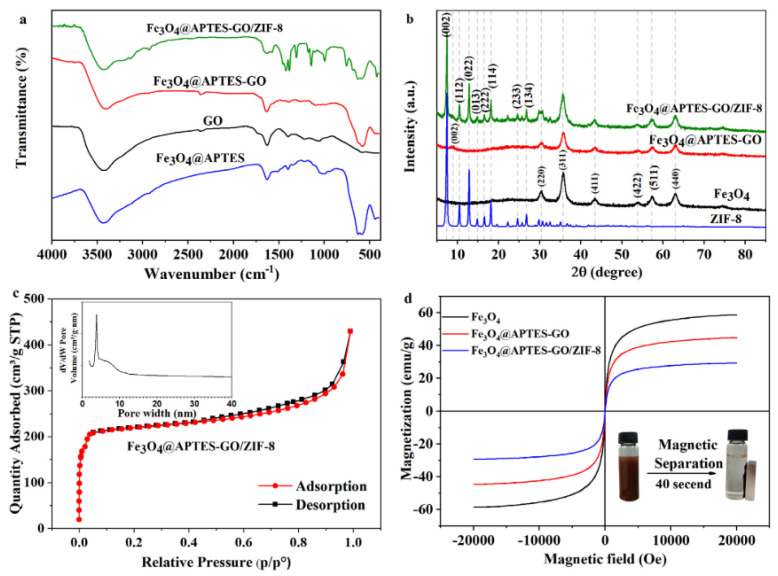
FT-IR spectra (**a**) and XRD patterns (**b**), the nitrogen sorption/desorption isotherms (**c**) and magnetization curves (**d**) of the prepared materials, presented by Senosy et al. [100].

The authors of another work [103] additionally used L-Cysteine to coat iron nanoparticles to increase the dispersion of Fe_3_O_4_ and increase the number of active centers on the surface of nanoparticles. In combination with the use of MIL-125-NH_2_ and UPLC-TUV, an analytical method was developed to detect compounds present in samples at such low concentrations as (LOD) from 0.05–0.2 μg/L, with recovery ranging from 83.8-109.4%, with an RSD lower than 8.9%. The prepared Fe_3_O_4_@Cys@MIL-125-NH_2_ is a promising magnetic adsorbent for efficiently enriching fluoroquinolones from the tap and environmental water [103]. Residues of organophosphorus pesticides in soil, water, and other agricultural products can cause neurotoxic and human diseases. Examples of such compounds include chlorpyrifos, profenofos, and phosalon. They have shown severe toxicity, mutagenic, carcinogenic effects and pose a considerable threat to mammalian life. An easy-to-apply, fast, and sensitive analytical method is required to analyze them. To extract such compounds from rice and water samples, a material called Fe_3_O_4_@TGA@TMU-6 was used [104]. The analysis was carried out using HPLC-UV. Due to the large surface area and unique porous structure of MOF, as well as π-π and hydrophobic interactions between analytes and MOF ligands, the prepared sorbent shows a high affinity for the target analytes. Under optimized conditions, the authors achieved LOD in water samples in the range of 0.5 to 1.0 μg/L for phosalon, chlorpyrifos, and profenofos. Residues of triazole pesticides pose similar threats, one of the largest groups of pesticides, which are key tools for controlling pathogens on several vegetables and fruits. Due to their high solubility in water and stability in the environment, they can enter food and drinking water [100]. It has also been proven that they can have a negative effect on organisms not targeted by humans. In this work [100], Fe_3_O_4_@APTES-GO/ZIF-8 material was characterized by FT-IR spectra, XRD patterns, nitrogen adsorption/desorption isotherms, and magnetization curves (Figure 16). The material was then used to determine selected fungicides. Under optimized conditions, in combination with the HPLC-DAD technique, an analytical method was developed suitable for detecting test compounds with LOD in the range of 0.014 to 0.109 μg/L, in the linearity range from 1 to 1000 μg/L with a relative standard deviation of 0.3 to 6.9%, and recoveries for the tested compounds in the range from 71.2 to 110.9%.

A summary of MOF materials used in MSPE is presented in Table 4.

### 3.5. Pipette-Tip Solid-Phase Extraction (PT-SPE)

One of the most significant advantages of the SPE technique is the possibility of multiple concentration of compounds present in the tested samples. One of the disadvantages of this technique is the need to use organic solvents in quantities ranging from a few to several mL per sample and the need to use a sorbent in an amount ranging from several dozen milligrams to several grams. Both of these disadvantages are contrary to the principles of green chemistry and affect the higher cost of analysis, especially when using such a unique sorbent like MOF. The pipette-tip solid-phase extraction (PT-SPE) technique is a miniaturized version of the SPE technique aimed at eliminating some of the disadvantages of the SPE technique [109]. It can be used in the process and with smaller amounts of organic solvents and sorbents, simply using the tip of the automatic pipette as a column for SPE and the automatic pipette itself as a source of vacuum or overpressure forcing the sample to flow through the sorbent bed. PT-SPE has become an effective tool in purifying, concentrating, and isolating specific analytes from the interferents present in the sample [110]. The PT-SPE technique is an excellent example of a method that uses fewer organic solvents, making it more economical and indirectly contributing to less environmental pollution [111]. One of the problems that can be encountered when using the PT-SPE technique is the same as in SPE: high back pressure, the resistance that the sorbent puts to the liquid sample flowing through it. This may be related to a densely packed solid sorbent in a relatively small “column” with a small internal diameter, which is particularly important when using an MOF, characterized by tiny pores. Another factor may be not using a vacuum collector capable of creating a significant vacuum but only the piston of the automatic pipette itself. This problem does not occur in other techniques and is also less common in the SPE technique. The solution may be to reduce the sorbent packing density, which will also help maintain the initial specific surface area and the sorption properties of the material itself. This effect can be achieved by using, as sorbents, composite materials created by combining MOF and polyacrylonitrile (PAN) nanofibers obtained by electrospinning. Electrospinning is a method of producing fibers of material with a diameter of several hundred nanometers. For this purpose, PAN is often used because it is characterized by adequate thermal, chemical, and mechanical resistance. Electrospinning is a simple, efficient method of producing continuous, porous fibers with a high specific surface-to-volume ratio. MOF/PAN structures, where MOFs are deposited on the nanofiber surface, are characterized by lower packing of MOF particles, preventing the problem of high back pressure [110,112]. An example of an electrospinning procedure for MOF/PAN nanofibers, in short: 0.48 g of UiO-66 in 3 mL DMF was dispersed using sonification, and 0.12 g PAN was added and mixed for 6 hours. After this time, another 0.2 g PAN was added to form a homogeneous, dispersed solution for electrical grading. The solution was subjected to a spinning process at 18 kV. The flow of the solution was 0.8 mL/h, and the diameter of the spine was 0.6 mm. The distance between the spindle and the collector was 20 cm. The procedure was carried out at room temperature. The resulting nanofibers were immersed in ethanol to exchange solvents and then dried overnight at 80 °C in a vacuum oven [110].

**Figure 17:**
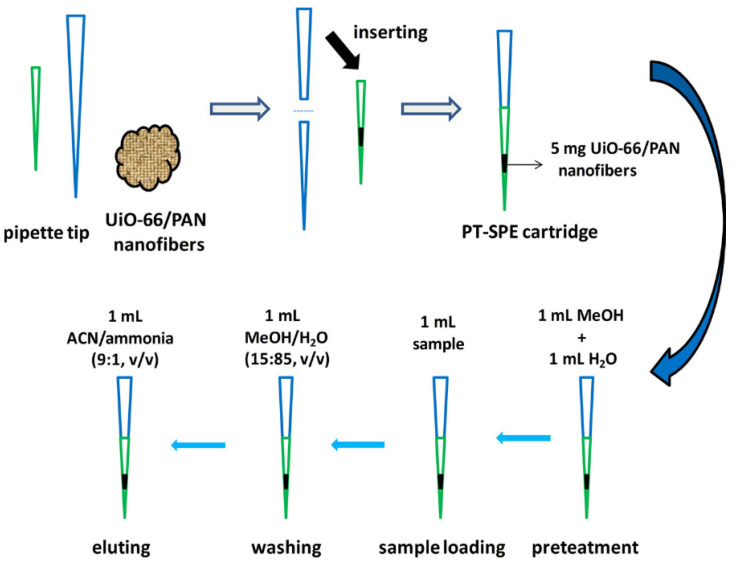
An example of a PT-SPE procedure, presented by Yan et al. [110].

Electrospinning in combination with MOF has been used in practice [111,112,113]. The use of the electrospinning method prevented the back pressure problem during the liquid sample flow through the tip of the pipette with a fine-grained sorbent of the MOF type. The most frequently appearing in MOF publications are again UiO-66 [110,113], MIL-68 [114], MIL-53(Fe) [111], and ZIF-8 [115]. MOFs are used in the PT-SPE technique to detect, among others, levofloxacin [116], bisphenol A [109], herbicides [110], and benzodiazepine drugs [112], in water [115], wastewater [116], food [114] and biological fluids [112]. The use of PT-SPE enables the use of a small amount of sorbent (even a few mg) and the testing of small sample volumes (<1 mL). In one of the studies [110], the authors used only 5 mg of UiO-66/PAN composite produced by electrospinning to analyze only 1 mL of liquid sample. Under optimized conditions, combined with the HPLC-FD technique, the authors were able to determine four selected phytohormones in samples of watermelon and mung bean tusks (Figure 17). The developed analytical method allowed for the detection of the investigated compounds with LOD at the level of 0.01 to 0.02 ng/mL. The linear ranges of the calibration plots were in the range of 0.06-60 ng/mL, with a correlation coefficient above 0.992. Recoveries for the analytes studied ranged from 84.4 to 111.2%, with the RSD in the range of 1.5–5.6%.

Fluoroquinolones (FQs) are commonly used to treat infections in humans and animals. These compounds are poorly absorbed by humans; they are usually excreted in urine or feces in unchanged form, where they end up in municipal wastewater. Wastewater treatment plants can be poor at removing them and they then end up back in the drinking water cycle like other pharmaceuticals and personal care products. Their detection at very low concentration levels can be carried out using the PT-SPE technique, using ZIF-8 as a sorbent [115]. The authors of this study used molecular simulations to elucidate further how ZIF-8 and fluoroquinolones interact. According to the calculations, the interactions between ZIF-8 and the tested compounds consisted mainly of hydrophobic interactions and hydrogen bonding between the oxygen/fluorine atom and the hydrogen atom of 2-methylimidazole. Under optimized conditions, the authors could detect compounds in LOD water samples ranging from 0.337 to 1.707 ng/L, with recovery from 75.9 to 96.8% from an RDR of less than 8.0%. Another example of drugs ending up in wastewater is benzodiazepines such as nitrazepam and oxazepam [112]. They are used to treat anxiety and sleep disorders, but their excessive consumption can lead to side effects such as memory loss and depression. The content of these compounds in wastewater samples is commonly detected using chromatographic techniques. However, the low concentration levels of these compounds in the samples and the complex matrix effectively hinder the analysis. A well-designed sample preparation step is necessary, leading to the concentration of analytes and removal of the influence of interferents. For this purpose, the authors of this study [112] used the PT-SPE technique, using a sorbent MIL-53(Al) combined with polyacrylonitrile in the process of electrical grading. In combination with the HPLC-DAD technique, under optimized conditions, the developed research method was successfully used to analyze these drugs in wastewater and biological fluids. LOD for the tested compounds ranged from 1.5 to 2.5 ng/mL; the linearity range of the method is 5.0–1000 ng/mL, with an RSD of <7.6% determined at three levels of analyte concentrations: 50, 100, and 250 ng/mL. Recoveries for the tested compounds were 92.4 and 100.3%.

A summary of MOF materials used in PT-SPE extraction is presented in Table 5.

### 3.6. Matrix Solid-Phase Dispersion (MSPD)

**Figure 18:**
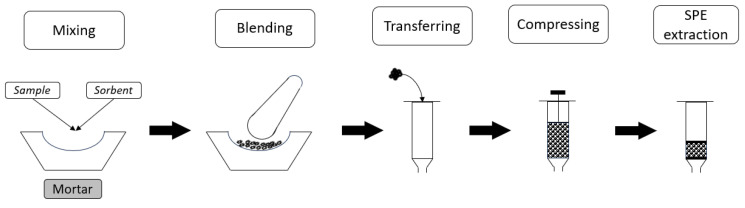
Application of the MSPD technique—variant [author’s own elaboration].

One of the lesser-known sample preparation techniques is a matrix solid-phase dispersion (MSPD). The first mention of this technique dates back to 1989, when Barker et al. described using it to isolate drug residues from tissues [121]. The concept of the technique itself is quite simple. It is designed for the analysis of solid and semi-solid samples. The sample and sorbent material are blended (for example, in a mortar) and then transferred into an empty SPE column or a syringe tube. The mixture of sorbent and sample is placed between two frits and then compressed (using a syringe-plunger) to avoid the formation of channels or air bubbles in the sorbent (Figure 18). The subsequent sample preparation steps are very similar to the typical SPE procedure. This technique ensures that the sample is completely broken down and dispersed into tiny particles, providing a better surface area for the subsequent sample extraction [122]. The sorbent used additionally acts as an abrasive for the sample during blending. Generally, a good compromise between a large surface area, good dispersion, and improved contact with the solvent is provided with the sorbents with a particle size of 40–100 µm. However, sorbents with a smaller particle size have also been used, although this leads to prolonged solvent elution times and possible blockage of the columns [123]. Since then, the technique itself has evolved considerably and increased the possibility of using both a range of classical [123] and modern nanomaterials, such as molecular-sieve-based sorbents, mesoporous materials, MOFs, molecularly imprinted polymers, and any others [124].

Due to the nature of this technique (the need to fill an empty column with a blended mixture of sample and sorbent), it is best suited for the analysis of solid, semi-solid, and viscous samples, such as biota, animal samples, and plants, but also sewage sludge and soil. MSPD is mainly used to analyze organic contaminants in environmental samples, for example, pesticides, herbicides, and fungicides in soil samples or pharmaceutical and cosmetic residues in plant samples [124,125]. Hoff et al., in their review article, described these problems and the most common applications of the MSPD technique, which are similar to the examples mentioned above [126]. The authors addressed the basic issues of MSPD but did not pay special attention to the applicability of modern sorbents, where this was mentioned in only one subsection. Another review article on MSPD does not focus directly on the technique itself but rather on the various ways to determine pharmaceuticals and cosmetics in sewage sludge, where MSPD is only one of several options for sample preparation [127]. Another review paper, written by Madej et al., once again focuses not on the MSPD technique itself but on its application to determining pesticides in fat-containing foods [128]. Having said that, the works mentioned may be a perfect choice for more target-oriented users. 

**Figure 19:**
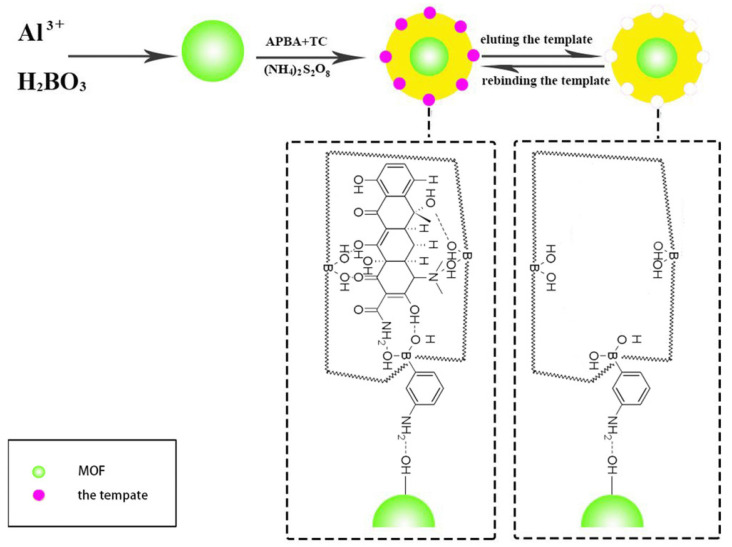
The synthesis process of MOF-MIP presented by Wang et al. MOF as a support material for the MIP synthesis [130].

To the best of our knowledge, there is no review article or at least a more comprehensive mention of the possibility of using MOFs as sorbents in MSPD. The most commonly used sorbents in the MSPD technique are classical sorbents such as C_8_, C_18_, or silica. They are characterized by high mechanical strength and versatility. On the other hand, MOFs exhibit a large specific surface area, and there is a possibility of adjusting their structure to increase the selectivity of the analysis. These advantages of MOFs, combined with molecularly imprinted polymer, were used by Liang et al. for the pyrethroids residue extraction from wheat [129]. The authors synthesized the sorbent used in the study and then characterized it using a series of instrumental techniques. The research methodology was successfully applied to detect residues of three pyrethroids in six wheat samples. After extraction, analysis was performed by gas chromatography-tandem mass spectrometry (GC-MS/MS). Under optimal conditions, the linearity range of pyrethroids in wheat samples was 10–1000 ng/g; detection limits were in the range of 1.8 to 2.8 ng/g, and accuracy (RSD) was less than 6.3%. MOFs do not have to be used only as the main sorbent; their applications go far beyond that. The other authors used MOF as a support material for the molecularly imprinted polymer synthesis for the tetracyclines in milk powder analysis. Tetracycline was used as a template molecule, and 3-aminophenyl boronic acid as a functional monomer and cross-linking agent (Figure 19). The new materials were then characterized using FT-IR, XRD, N_2_ adsorption/desorption measurements, and others. The extraction was followed by ultra-high-performance liquid chromatography with tandem mass spectrometry. Under the optimal conditions, the recoveries were in the range of 84.7–93.9%, with the detection limits of the tetracycline in the range of 0.217–0.318 ng/g [130].

**Figure 20:**
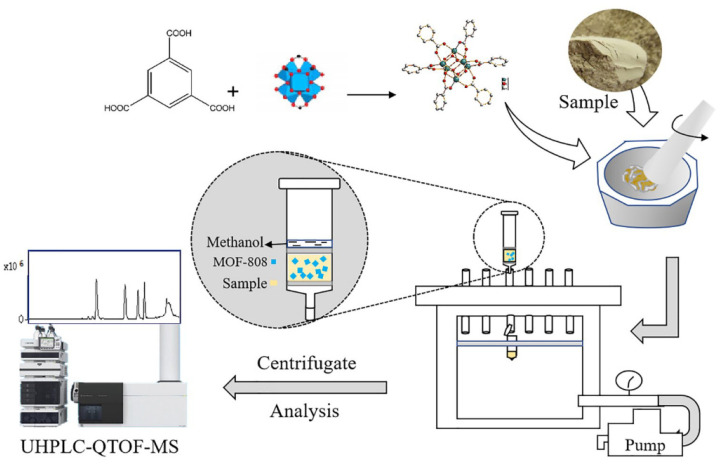
The synthesis strategy of MOF-808 and extraction process of the developed method are presented by Zhang et al. [133].

The most common applications of the MSPD technique include the analysis of pesticides in plant samples. This is how the authors of one study [131] used the MOF, synthesized based on lanthanides, to analyze pesticides (atrazine, bifenthrin, bromuconazole, clofentezine, phenbuconazole, flumethrin, procymidone, and pyrimicarb) from bell pepper (Capsicum annum L.). After extraction, gas chromatography coupled to mass spectrometry was used in selected ion monitoring mode. The developed method was linear in the range of 50.0–1000.0 µg/kg for procymidone and 200.0–1000.0 µg/kg for the other pesticides. Correlation coefficients ranged from 0.9930 to 0.9992, and substance recoveries ranged from 52.7 to 135.0%, with CV values between 5.2 and 5.4%. Using MOF as a sorbent, it is possible to obtain excellent results from quantitative analysis [131]. An equally interesting paper comparing the sorption properties of several selected nanomaterials as sorbents was published by Rodriguez-Ramos et al. [132]. The authors compared the use of graphene oxide, multi-walled carbon nanotubes, and 1,3,5-benzenetricarboxylate iron MOF as sorbents in the MSPD technique, with the latter showing the best performance in terms of extraction and purification efficiency of samples of fifteen phthalates from various environmental samples, prior to their separation and quantification by ultra-high performance liquid chromatography coupled to triple quadrupole mass spectrometry. The entire methodology was validated for agricultural soil and sand. The method’s limits of quantification ranged from 0.14 to 2.7 µg/kg dry weight, with recoveries ranging from 70 to 120% and RSD below 20% [132]. The authors of another study [133] used a Box-Behnken design to optimize the experimental conditions for MSPD microextraction of five saponins in leaves (Figure 20). A simple MOF was used to obtain good linearity of >0.998 in the range of 0.01–100 µg/ml, with recoveries ranging from 87.04 to 103.78% and RSD lower than 5%. Compared with the traditional extraction method, the new MOF-assisted MSPD showed overall better performance, with low consumption of organic reagents [133].

A very interesting paper was presented by Souza et al [134]. The research team tested the feasibility of using MOFs as a sorbent in the MSPD technique to analyze pesticide residues in chicken (Gallus gallus domesticus) egg samples. Two MOFs were tested as adsorbents in MSPD to extract lindane, bifenthrin, and pyrimicarb from lyophilized chicken eggs. A validated methodology was used to evaluate the performance of the new materials. The MOFs showed satisfactory results compared to conventional materials. More importantly, the MOFs allowed for the use of less sorptive material than conventional sorbents. After extraction, GC-MS was performed in the monitoring mode for selected ions. Analytical curves of 0.01–2 µg/ml showed good linearity (>0.999), with recoveries in the range of 70–120%, with RSD below 7.9% [134].

A summary of MOF materials used in MSPD is presented in Table 6.

### 3.7. Stir-Bar Sorptive Extraction (SBSE)

The first mention of SBSE dates back to 1999, and an article was published by Baltussen et al [135]. The authors of the article developed a new, for their time, technique using the PDMS sorbent, which is still popular to this day. SBSE was a response to the inadequacies of the then-used open tubular trapping (OTT) technique and a competitor to the more recently developed SPME. The initial approach to the technique was to remove a layer of Teflon from the magnetic core of a magnetic stirrer, then place it in a glass tube and cover it with a layer of PDMS (polydimethylsiloxane) sorbent. The device prepared in this way was placed in a liquid sample and subjected to stirring for a specified time. The size of the magnetic stirrer and the amount of PDMS sorbent used can be adjusted depending on the amount of sample. In the first article, the authors used thermal desorption of analytes, although desorption using organic solvents and liquid desorption is equally possible [135]. The SBSE technique appears to be an extremely interesting technique for several reasons, including the ubiquity of magnetic stirrers, which can be easily found in almost any laboratory, which significantly reduces the cost and complexity of using the technique in practice, the relatively large amount of sorbent material used, which can increase the sensitivity of the analyses conducted, and the ease of controlling the length of extraction time, which, due to the direct contact of the sorbent with the sample, can be very efficient.

Since the publication of the first historical article, the SBSE technique, like all others, has undergone significant development. However, to this day, still the most popular sorbent used in the SBSE technique is polydimethylsiloxane. It is excellent for extracting non-polar compounds from aqueous matrices and is mainly used for this purpose. The most typical applications of SBSE include the extraction of PAHs from environmental matrices, foodstuffs, and biological samples. In addition to it, commercially, you can only get two other types of sorption coatings, polyethylene glycol (PEG) and polyacrylate (PA), which are better for the extraction of more polar compounds. However, the extraction of highly polar and hydrophilic analytes from these matrices remains a challenging issue. A review article published by Hasan et al. [136] describes these problems and focuses on a comparison of different application methods (adhesion, sol-gel, MIP, monolithic) of modern sorption materials, such as nano- and micro-carbon-based materials, functional polymers, and inorganic nanoparticles, to change the selectivity of the stir bar to more polar compounds. Although MOF is mentioned, it is very insignificant [136]. Another review article published by Manousi et al. [137] does not focus on changing the selectivity of the stir bar but on the use of various modern nanomaterials, including MOFs, in the most typical application of this technique, namely the extraction of PAHs from environmental samples. Here, too, the most common MOFs are MIL-100(Cr), MIL-100(Fe), and ZIF-7(Zn) [137]. In relation to the latter, a lot of interesting information can be found in the article by Rodas et al. [138], which focuses exclusively on using ZIF-type materials, omitting the whole range of the rest of the MOFs.

**Figure 21:**
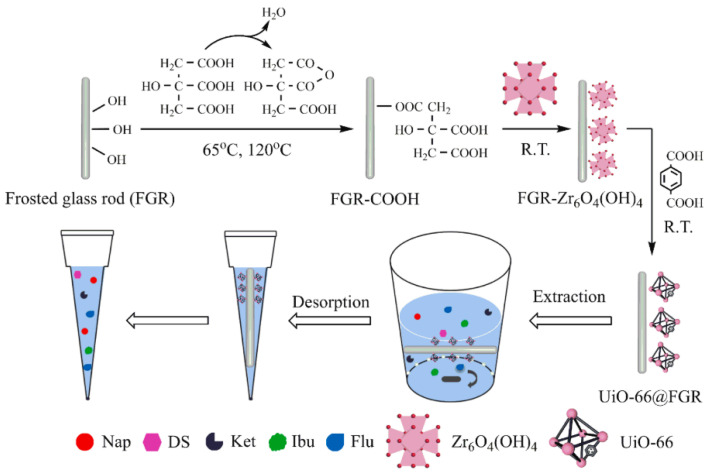
UiO-66@FGR application to extract selected NSAIDs, presented by Zhang et al. [140].

An interesting approach to using MOFs was taken by Khoobi et al. [139] with in-situ growth of ZIFs on the surface of layered double hydroxides (LDHs) for the preparation of a nanosorbent used in stir-bar sorptive extraction. The designed nanosorbent was used to extract and detect benzylpenicillin in biological and food samples. Under optimized conditions, the limits of detection and quantification were 0.05 and 0.15 µg/L, respectively [139]. UiO-66, one of the most known and durable MOFs, has been used as a component for a sorbent by Zhang et al. [140] to extract and detect 5 non-steroidal anti-inflammatory drugs (ketoprofen, flurbiprofen, ibuprofen, naproxen, and diclofenac sodium) in sheep muscle, chicken wing, and milk. UiO-66 was prepared on a frosted glass rod (FGR) through the coordination interaction of Zr-OH groups and carboxyl sites on FGR (Figure 21). The optimized method, coupled with UHPLC, showed excellent results, giving another example in favor of using MOFs as modern sorbents [140].

Another example of using MOFs as sorbents in MSPE technology is based on the use of amino-modified MOF (NH_2_-MIL-101(Al)) coating on monolith onto a conventional Teflon-coated magnetic stir bar [141]. The external surface of the Teflon stir bar was first vinylized to immobilize a glycidyl methacrylate (GMA)-based polymer onto the magnet. Then, MOF was covalently attached to the GMA-based monolith. The designed stir bar was evaluated as an SBSE sorbent for the extraction of three estrogens (estrone, 17β-estradiol, and estriol) and also synthetic 17β-ethinylestradiol from water and human urine samples. Researchers obtained satisfactory detection limits in the range of 0.015–0.58 µg/L, with recoveries ranging from 70 to 95% [141].

A summary of MOF materials used in SBSE is presented in Table 7.

## 4. Limitations in the Application of MOFs

Despite their many advantages, MOF materials also have several disadvantages. Some are related to the synthesis of these materials and include: a long reaction time, high temperature requirement [144], reaction solvent [145] or problems with obtaining a large amount of product [146]. Additionally, MOFs may be characterized by low chemical and thermal stability and sensitivity to contamination and cyclability issues, hindering their practical applications [147]. MOFs may also face disadvantages like mass transfer limitations, especially through crystallite surfaces, which may affect their uptake rates and performance in adsorptive separations and catalysis [148]. The powdered state of MOFs often results in difficulties with transfer and separation, as well as with recovery procedures, which reduces their adsorption properties [149]. Another important aspect is the instability of many MOFs in air, water, and acid-base solutions, which limits their further commercial application and means it is not possible to realize large-scale production [150]. There is also a concern about MOF toxicity in H_2_O, as the washing out of adsorbents can lead to contamination which is greater than the initial pollution [149,151]. The increasing cost of organic ligands used to prepare MOFs is another drawback [152]. All these limitations may reduce the applicability of MOF materials in various sample preparation techniques.

## 5. Conclusions

Many MOF materials are relatively unstable in an aqueous environment where they are hydrolyzed and subsequently degraded. The stability of MOFs in contact with water depends on many factors, one of which is the strength of the coordination bond connecting the organic ligand and the metallic node, while another is the steric obstacle that the ligand may pose to prevent water molecules from coming into contact with the reactive node. Currently, there are already several MOFs that show an increased resistance to water, and their sorption properties allow them to be used in various techniques for sample preparation for analysis. Among them, we can mention those belonging to the “families” ZIF—Zeolitic Imidazolate Framework, UiO—University of Oslo, and MIL—Materials from Institut Lavoisier. These MOFs, due to their properties, are most often used by many researchers in their studies.

One of the advantages of MOFs is the possibility of modifying them in a wide range, adapting their sorption properties to a specific application, and increasing sorption efficiency. Theoretically, it is possible to synthesize many types of MOFs, differing in the kind of ions or clusters of metals and organic connectors. In practice, only a few dozen MOFs are used. This is due to the difficulties encountered in the process of their synthesis or their price. Not every planned structure of MOF is possible to implement in practice, and the process of their synthesis itself depends on many factors, such as the type of solvent, type of substrates, concentration of substrates, presence of organic bases, type of counterions, temperature, mixing rate, etc. Because of these difficulties, in laboratory practice, few researchers deal with the matching of MOFs, either through directed synthesis or through the modification of the existing structure, in order to improve the sorption properties and to produce the sorbent thus obtained to a specific compound or group of compounds. The observed direction of development of these studies is usually based on the simple use of already available MOFs to investigate their general sorption properties (common organic pollutants present in the environment and possible modification of the sample preparation process for analysis). Although the modification of the structure of the MOF itself is an unpopular direction of work on their development, there is a clear tendency to create composite materials combining the advantages of MOFs with the advantages of other materials. The ingenuity of researchers in creating such composite materials is extremely high: cellulose aerogels, nanofibers, cotton, composites of the Fe_3_O_4_/TEOS/APTES type, graphene oxide, chitosan, and porous carbons. Often, the composite material created is aimed at adapting the material to be used in a specific sample preparation technique (MOF@Fe_3_O_4_ in MSPE). Various techniques are used to create new composite materials containing MOFs, giving the obtained materials a unique structure and properties, to eliminate the disadvantages of a given sample preparation technique, such as the use of electrospinning to get MOFs deposited on nanofibers, which eliminates the problem of high back pressure in the PT-SPE technique. Combining MOFs with another material could also positively affect their durability in the aquatic environment.

Analyzing emerging scientific publications, it can be seen that another trend in using MOFs as sorbents for the analysis of liquid samples is the miniaturization and development of techniques such as D-SPE, μ-SPE, MSPE, and PT-SPE. Due to the properties and high specific surface area of MOFs, it is possible to use these materials in micro-quantities without losses on the recovery and detection limit of the determined compounds. The use of smaller amounts of organic solvents and smaller amounts of sorbents translates into more significant savings for a given laboratory and is in line with the principles of green chemistry. The use of sorbents in an unusual way, such as in the μ-SPE technique, allows for the preparation of samples with complex matrices.

A small number of research laboratories carry out research focused on the durability of the materials they obtain. An additional test carried out after the synthesis of MOFs, in addition to checking, for example, the specific surface area, should be checking this surface after contact of the MOF with water or checking the recoveries of the tested compounds determined with the use of a single column repeatedly. These studies will help to estimate the durability of MOFs in various sample preparation techniques and, in practice, will help determine how many times, for example, a selected SPE column containing an MOF sorbent can be reused before too small recoveries or too large fluctuations in relative standard deviation determined for subsequent analyses are observed.

In summary, MOF materials are widely used in various sample preparation techniques and are an attractive alternative to currently used sorption materials. Additionally, the use of these materials increases recovery and lowers detection limits, so the potential use of MOFs at the sample preparation stage is still growing and has great potential.

## Figures and Tables

**Table 1:** Selected examples of using MOFs as sorbents in the SPE technique.

MOF	Analytes	Matrix/Sample	Analytical Technique	LOD	LOQ	Linearity Range	R^2^	Recovery	RSD	Source
MIL-101(Cr) @ Graphene hybrid aerogel	5 non-steroidal anti-inflammatory drugs (NSAIDs)	Deionized water, Tap water	HPLC-UV-Vis	0.01–0.10 ng/mL	–	–	>0.9973	89.2–100.7%	3.7–8.5%	[33]
MIL-101(Cr) and MIL-100 (Fe)	4 sulphonamides	Environmental water	UPLC-MS/MS	0.03–0.08 μg/L	0.11–0.27 μg/L	0.2~40 or 0.5~100 μg/L	>0.996	83.5–107.3%	0.2–8.0%	[34]
μMNCs derived from ZIF-67	Anionic surfactants	Environmental water (natural water, groundwater, wastewater)	CCD spectrophotometer	100 μg/L	–	50–1000 μg/L	0.998	93–110%	2.7%	[35]
MIL-101	11 UV filters compounds	Environmental water	GC-MS/MS	1.0–11.7 ng/L	–	0.5–100 μg/L	≥0.9973	82–105%	<10%	[37]
UiO-66 and UiO-66-NH_2_	7 substituted phenols	Groundwater	HPLC-UV-Vis	0.1–0.2 μg/L	–	0.5–200 μg/L	0.990–0.999	90–98%	4.7–5.7%	[38]
Chitosan/MIL-68(Al)	Pb^2+^, Cd^2+^	Gastropods	ICP-OES	0.16 μg/L	0.5 μg/L	0.5–50 μg/L	0.9984–0.9976	95.0–97.5%	3.8–4.3%	[39]
MOF-5, MOF-235, UiO-66(Zr)-2 OH	PFCs and PFOS	Water samples	MALDI-TOF-MS	0.64–0.94 ng/mL	–	5–1000 ng/mL	0.996–0.999	92.8–103%	0.34–12%	[40]
UiO-66-NH_2_@MIP	4 aflatoxins AFB1, AFB@, AFG1, AFG2	Grain	HPLC-MS	60–130 ng/kg	240–450 ng/kg	0.20–45 μg/kg	0.9986–0.994	74.3–98.6%	1.0–5.9%	[41]
CD-MOF	5 sulfonamides (SAs)	Meat samples (Chicken, pork, liver, and fish)	HPLC-UV	0.32–2.0 ng/mL	–	10–1000 ng/mL	0.9907–0.9995	76–102%	2.4–6.5%	[42]
CH_3_MOF-5/PAN	2 estrogenic drugs (levonorgestrel, megestrol acetate)	Urine samples	HPLC-UV-Vis	0.02 μg/L	0.07 μg/L	0.05–100 μg/L	>0.999	82.8–94.8%	2.8–4.1% and 2.5–4.2%	[43]
MOF-5	PAHs	Environmental water	HPLC-FLD	0.4–4.0 ng/L	–	0.004–20 μg/L	>0.996	80.2–120.2%	0.5–11.7%	[44]
CS/MOF-SH	Pb^2+^, Cd^2+^	Food samples	GF-AAS	0.033 μg/L and 0.008 μg/L	–	0.1–100 μg/L and 0.01–10 μg/L	0.996 and 0.998	–	3.9–4.1%	[45]
TMU-6	3 Plasticizer (DBP, DEHP, DOA)	Bottled water	GC-FID	0.2–0.7 μg/L	–	0.5–100 μg/L	>0.994	88–110%	≤6.3%	[46]
DMP@HKUST-1	Hg^2+^	Rice	AFS	0.0125 ng/mL	–	–	–	98.8–109%	<6%	[47]
MOF-199(Cu)	Non-steroidal anti-inflammatory drugs (NSAIDs)	Human plasma and water samples	HPLC-UV	0.01–0.02 and 0.03–0.1 ng/mL	–	0.03–300 ng/mL and 0.1–200 ng/mL	0.9938–0.9989	93.6–99.6%	3.5–5.1%	[48]

**Table 2:** Selected examples of using MOF as sorbents in the SPME technique.

MOF	Analytes	Matrix/Sample	Analytical Technique	LOD	LOQ	Linearity Range	R^2^	Recovery	RSD	Source
UIO-66/MoS_2_	Polycyclic Aromatic Hydrocarbons (PAHs)	Fish samples	GC-MS	0.11–1.4 ng/kg	0.36–4.61 ng/kg	0.5–10,000 ng/kg	0.988-0.997	80.2–101%	<6%	[52]
Cu-BDC//polyimide composite	Polycyclic Aromatic Hydrocarbons (PAHs)	Water samples	GC-MS	0.11–2.10 ng/L	0.36–6.99 ng/L	20–5000 ng/L	0.9957-0.9976	81.7–116%	4.2–12.7%	[53]
MOF-74 derived porous carbon	Odorous organic contaminants	Tap water, freshwater, and wastewater effluent	GC-MS	0.00001–0.1 μg/L	0.00003–0.3 μg/L	0.005–100 μg/L	0.993-0.999	83.6–115.5%	<9.4%	[54]
MIL-88(Fe)/GO	Phthalic acid esters (PAEs)	Vegetable oils	GC-FID	0.5–2.0 ng/g	1.7–6.7 ng/g	1.7–500 ng/g	>0.994	83.1–104.1%	<10.5%	[55]
MOF-199/GO	8 organochlorine pesticides (OCPs)	River water, soil, water convolvulus, and longan	GC-ECD	2.3–6.9 ng/L	–	0.01–1.0 μg/L	0.9948–0.9993	72.2–104.4%	5.3–8.8%	[56]
Fe_3_O_4_@MIL-101(Cr)/PANI nanocomposite	Endogenous aldehydes (hexanal and heptanal)	Human plasma and urine samples	GC-FID	0.001 and 0.01 μg/L	–	0.01–1 and 0.1–1 μg/L	–	98.5–113.5% and 95.2–122.1%	3.5–7.1% and 10.4–15.7%	[57]
UiO-66-OH	Polybrominated diphenyl ethers (PBDEs)	Milk	GC-MS/MS	0.15–0.35 ng/L	–	1.0–500 ng/	>0.9994	74.7–118.0%	7.58–9.48%	[58]
UMCM-1, MOF-DES/MIPs	Phthalate esters	Yogurt, water, and soybean oil	GC-FID	0.008–0.03 μg/L	0.028–0.12 μg/L	0.01–1000 μg/L	0.996–0.998	95.5–100.0%	2.4–4.7%	[59]
ZIF-8-monolith composite	Fluoroquinolones (FQs)	Environmental waters and honey samples	HPLC-FLD	0.14–1.1 ng/L	0.48–3.87 ng/L	0.001–10 μg/L	0.9983–0.9999	80.1–119%	<10%	[60]
CIM-80(Al)	6 volatile methylsiloxanes and 7 musk fragrances	Environmental water samples	GC-MS	0.2–0.5 μg/L	–	0.2–200 μg/L	>.996	61.4–145%	<17%	[61]
MIL-53(Al, Cr, Fe)	16 Polycyclic aromatic hydrocarbons (PAHs)	Water samples	GC-MS/MS	0.10–0.73 ng/L	–	1–500 ng/L	>0.98	70–125%	<12.5%	[62]
MIL-88B	Polychlorinated biphenyls (PCBs)	Water and soil samples	GC-MS	0.45–1.32 ng/L	–	5–200 ng/L	0.990–0.999	79.7–103.2%	4.2–8.7%	[63]
UiO-67	Nitrobenzene compounds	Water	GC-MS	5.0–10.0 ng/L	–	0.015–12 μg/L	0.9945–0.9987	74.0–102.0%	<11.9%	[63]
MIL-101(Cr)	BTEX and PAHs	Water samples	GC-MS	0.32–1.7 and 0.12–2.1 ng/L	–	10–20,000 and 10–500 ng/L	0.9914–0.9996	80.0–113 and 84.8–106%	<7.7%	[64]
UiO-66	Phenols	Water samples	GC-FID	0.11––1.23 μg/L	–	1–1000 μg/L	0.993–0.999	80–115%	2.8–6.2%	[65]

**Table 3:** Selected examples of using MOF as sorbents in the D-SPE and μ-SPE techniques.

Sample Preparation Technique	MOF	Analytes	Matrix/Sample	Analytical Technique	LOD	LOQ	Linearity Range	R^2^	Recovery	RSD	Source
D-SPE	Core-shell indium (III) sulfide@MIL-125(Ti)	16 Nitro-PAHs	Water samples	GC-MS/MS, NCI	2.9–83.0 ng/L	9.80–120 ng/L	10–1000 ng/L	>0.99	71.3–112.2%	<10%	[69]
D-SPE	MIL-101(Cr)	Benzo(a)pyrene (BaP)	Edible oil	HPLC-FLD	0.19 ng/mL	0.434 μg/L	1–30 ng/mL	0.9958	88.8–118.8%	1.07–8.14%	[70]
D-SPE	MIL-100(Cr), MIL-100(Fe) and MIL-53(Al) 7:1:2	Tetracyclines (TCs)	Honey	HPLC-MS/MS	0.073 to 0.435 ng/g	0.239–1.449 ng/g	0.25–500 ng/g	0.9965–0.9990	88.1–126.2%	4.3–9.4%	[71]
UA-SPE	Chitosan/MIL-53(Al) foam	5 Parabens	Environmental water and drinking water	UPLC-MS/MS	0.09–0.45 μg/L	–	0.5–200 μg/L	0.9948–0.9983	78.75–102.1%	<7.4%	[72]
μ-SPE	UiO-66(Zr)	4 Androgens and Progestogens	Environmental water	LC-MS/MS	2.0–10.0 ng/L	7.0–20.0 ng/L	7.624–2032 ng/L	0.9990–0.9997	80.5–102.4%	<10.0%	[73]
D-SPE	UiO-67	Sulfonamides (SAs)	Meat samples (chicken, lamb, beef)	HPLC-DAD	0.7–6.5 ng/g	–	14.6–250 ng/g	≥0.9991	83.4–103.8%	3.4–4.7%	[74]
D-μ-SPE	MIL-101(Cr)-NH_2_	Progesterone	Lake water and synthetic urine	DART-MS	0.02 ng/mL	0.07 ng/mL	0.5–500 ng/mL	0.9992	92.0–117.8%	2.4–8.4%	[75]
USA-IL-μ-SPE	[Zn_2_(BDC) _2_ (DABCO)]_n_ and ([BMIM] [PF6])	Hg^2+^	Human serum	CV-AAS	6.5 ng/L	–	0.02–5.5 μg/L	0.9988–0.9992	96–105%	4.2%	[76]
VA-SPE	MIL-101(Cr)/PVA cryogel	4 non-steroidal anti-inflammatory drugs	Environmental water	HPLC-MS/MS	0.007–0.037 μg/L	–	0.10–10 μg/L and 0.0020–2.0 μg/L	≥0.9934	78.44–105.7%	1.33–9.85	[77]
UAE-μ-SPE and VA-μ-SPE	MIL-101(Cr)	Polar estrogens	Water samples	UPLC-MS/MS	0.95–23 ng/L	3.74–22.5 ng/L	5–100.000 ng/L	0.996–0.999	85.4–120.8%	≤9.9%	[78]
D-μ-SPE	UiO-66-NH_2_	Chlorophenoxy acids herbicides (CPAHs)	Biosamples, vegetables	HPLC-MS	0.16–0.37 ng/g	0.46 to 1.15 ng/g	10–1000 pmol/mL	>0.9985	82.3–102%	3.1–5.9%	[79]
D-μ-SPE	M-MIL-53(Fe)	4 Phenols and anilines	Environmental water	HPLC-PDA	0.03–0.2 μg/L	–	0.1–2000 μg/L	0.9992–0.9995	39.5–93.3%	3.5–12.6%	[80]
VA-D-μ-SPE	HKUST-1	7 Parabens	Drinking and pool water, Cosmetic creams, Human urine	HPLC-DAD	1.5–2.6 μg/L	5.0–8.7 μg/L	0.5–147 μg/L	>0.9966	80.3%	<10%	[81]
VA-D-μ-SPE	IL-MIL-100(Fe)	PAHs	Environmental water, vegetable, and fruit juice	GC-FID	2.0–5.5 ng/L	6.1–16.8 ng/L	0.02–200 ng/mL	0.9984–0.9997	97–103.5%	3.0–3.8 and 4.1–4.9%	[82]
VA-D-μ-SPE	MIL-101(Cr)	5 nitroimidazole residues	Environmental water	UPLC-MS/MS	0.03–0.06 μg/L	0.09–0.20 μg/L	0.1–20 and 0.2–40 μg/L	>0.992	75.2–98.8%	<8%	[83]
MAE-D-μ-SPE	MIL-101 (Cr)	Herbicides	Soybeans	HPLC-DAD	1.56–2.00 μg/kg	5.20–6.68 μg/kg	5.00 to 513 μg/kg	0.9996–0.9998	91.1–106.7%	≤6.7%	[84]
MA-D-μ-SPE	MIL-101(Cr)@GO	The pharmaceutical residue (MNZ, TNZ, CAP, SMX)	Chicken breast	HPLV-MS/MS	0.08–1.02 ng/kg	0.26–3.40 ng/kg	1–100 ng/kg	≥0.9928	88.9–102.3%	2.5–4.3%	[85]
MD-μ-SPE	GO/MOF-74/Fe_3_O_4_/PTy	Prokinetics drugs (DOM and ITP)	Human plasma	HPLC-UV	0.4–1.1 ng/mL	–	1.5–1100 ng/mL and 4.0–1750.0 ng/mL	0.991–0.995	88.0–90.0%	8.6–9.0%	[86]
μ-SPE	ZIF-8	6 PAHs	Environmental water	GC-MS	0.002–0.012 ng/mL	0.029–0.083 ng/mL	0.1/0.5–50 ng/mL	0.9955–0.9995	98.0–106.5%	<8.5%	[87]
μ-SPE	MIL-101	5 organochlorine pesticides	Water	GC-MS	0.0025–0.016 ng/mL	0.010–0.074 ng/mL	0.05–50 ng/mL	>0.9946	87.6–98.6%	<10%	[88]
μ-SPE	MIL-101(Cr)	6 Phthalate esters	Drinking water	GC-MS	0.004–0.02 μg/L	0.01–0.07 μg/L	0.1–50 μg/L	>0.9942	71.5–93.5%	0.8–10.9%	[89]
μ-SPE	HF-MIL-101	7 PCBs	Environmental water	GC-MS/MS	0.15–0.63 ng/L	0.51–2.07 ng/L	5–1000 ng/L	0.991–0.996	83.0–115.9%	<13.5%	[90]

**Table 4:** Selected examples of using MOF as sorbents in the MSPE technique.

MOF	Analytes	Matrix/Sample	Analytical Technique	LOD	LOQ	Linearity Range	R^2^	Recovery	RSD	Source
Fe_3_O_4_@MOF-808	Benzoylurea insecticides (bus)	Tea beverages and juice samples	HPLC-DAD	0.04–0.15 ng/mL	0.15–0.50 ng/mL	0.15–50 ng/mL	09971–0.9999	84.6–98.3%	2.6–11.4%	[36]
ZIF-7	PAHs	Rainwater	GC-MS	0.71–5.79 ng/L	2.50–19.2 ng/L	0.05–5 ng/mL	>0.9944	>82%	<9.2%	[91]
Fe_3_O_4_@SiO_2_-MIL-101(Cr)	PAHs	Environmental water	HPLC-PDA	2.8–27.2 ng/L	6.3–87.7 ng/L	–	–	81.3–105%	3.1–8.7 and 6.1–8.5%	[96]
Fe_3_O_4_/ZIF-8/IL	4 Pyrethroid insecticides	Tea infusions	GC-MS/MS	0.0065–0.1017 μg/L	–	0.5–50 and 0.5–500 μg/L	>0.999	72.1–96.8%	9.70–11.95%	[97]
Fe_3_O_4_@SiO_2_@Ti-MOF	Caffeic acid (CA)	Medical extracts of plants and water samples	UPLC-UV	0.016–0.021 ng/mL	0.052–0.068 ng/mL	0.15–3200 ng/mL	–	99.76%	1.84–5.54%	[98]
Fe_3_O_4_@SiO_2_-GO/MIL-101(Cr)	Seven triazine herbicides	Rice samples	HPLC-UV-Vis	0.010–0.080 μg/kg	0.050–0.28 μg/kg	2.00–1000.00 μg/kg	0.9992–0.9998	83.9–103.5%	< 8.7%	[99]
Fe_3_O_4_@APTES-GO/ZIF-8	Four triazole fungicides	water, honey, and fruit juices	HPLC-DAD	0.014–0.109 μg/L	0.047–0.365 μg/L	1–1000 μg/L	≥0.9914	71.2–110.9%	0.3–6.9%	[100]
Fe_3_O_4_@MOF-5	Heterocyclic pesticides (Carbendazim, Triadimefon, Chlorfenapyr, Fenpyroximate)	Environmental water	HPLC-DAD/FLD	0.04 μg/L and 0.13 μg/L	0.11 μg/L and 0.35 μg/L	0.3–500 μg/L and 0.1–500 μg/L	>0.9992	80.20–108.33%	2.98–7.11% and 3.31–7.12%	[101]
Fe_3_O_4_@MIL-100(Fe)	Seven NSAIDS	Environmental water	UPLC-MS/MS	0.02–0.09 μg/L	0.06–0.3 μg/L	0.1–10 μg/L 0.2–20 μg/L and 0.3–30 μg/L	0.9987–0.9995	75.2–105.2%	≤9.6%	[102]
Fe_3_O_4_@Cys@MIL125-NH_2_	Five fluoro- quinolones	Water samples (Tap and river water)	UPLC-TUV	0.05–0.2 μg/L	0.1–0.5 μg/L	0.1–1000 μg/L	0.9950–0.9995	83.8–109.4%	<8.9%	[103]
Fe_3_O_4_@TGA@TMU-6	Organophosphorus pesticides	Rice and environmental water	HPLC-UV	0.5–1 μg/L	7.5–10.0 μg/L	7.5–75 μg/L 10–100 μg/L and 10–150 μg/L	0.9912–0.9987	88.0–107.2%	4.8–7.1%	[104]
Fe_3_O_4_@SiO_2_-MOF-177	Phenols	Environmental water	GC-MS	16.8–208.3 ng/L	56.0–694.2 ng/L	1–200 μg/L	–	83.3–108.7%	4.2–8.5% and 5.1–9.2%	[105]
MG@MIL-100-B	Endogenous catecholamines (DA, E and NE)	Rat plasma	HPLC-MS/MS	0.005–0.02 ng/mL	0.01–0.1 ng/mL	0.01–2 and 0.10–8 ng/mL	0.9909–0.9943	94.40–109.51%	2.84–11.44%	[106]
MOF-5@ Fe_3_O_4_	Fluoroquinolones (FQs)	Milk	HPLC-MS/MS	0.009–0.016 ng/mL	–	0.5–1000 ng/mL	0.9965–0.9995	95.33–104.7%	2.93–4.21%	[107]
MIL-100(Cr)	Malachite green (MG)	Drinking, tap, Groundwater, Trout fish	HPLC-UV-Vis	0.012 mg/L	0.040 mg/L	0.04–2 mg/L	0.9980	95–107%	1.4–4.3%	[108]

**Table 5:** Selected examples of using MOF as sorbents in the PT-SPE technique.

MOF	Analytes	Matrix/Sample	Analytical Technique	LOD	LOQ	Linearity Range	R^2^	Recovery	RSD	Source
Th-MOF	Bisphenol A	Bottled drinking water	HPLC-FD	0.0010 ng/mL		0.02–0.456 ng/mL	0.996	93.3–106.7%	3.2–5.0%	[109]
UiO-66/PAN nanofibers	4 Phytohormones	Watermelon and mung bean sprouts	HPLC-fluorescence detector	0.01–0.02 ng/mL	–	0.06–60 ng/mL	>0.992	84.4–111.2%	1.5–5.6	[110]
Mil-53(Fe)/PAN nanofibers	Benzodiazepine drugs	Wastewater and biological fluids	HPLC-DAD	1.5–2.5 ng/mL	–	5.0–1000 ng/mL	0.9991 and 0.9995	92.4–100.3%	≤7.6%	[112]
Cotton@UiO-66	4 Phenoxy Herbicides	Soil, cucumber, tap water	HPLC-PDA	0.1–0.3 μg/L	0.3–1.0 μg/L	1.4–280 μg/L	0.9959–0.9996	83.3–106.8%	<6.3%	[113]
MIL-68@COF	Sulfonamides	Water, milk, and meat samples	HPLC-VWD	1 ng/mL	–	10–2000 ng/mL	0.9987–0.9998	68.9–103.8%	<5.2%	[114]
ZIF-8/cellulose aerogel	Fluoroquinolones (FQs)	Bottle, tap, and river water samples	HPLC-FLD	0.337–1.707 ng/L	1.012–5.120 ng/L	1.0–512.0 ng/L	0.9954–0.9992	75.9–96.8%	<8.0%	[115]
Co-MOF	Levofloxacin	Wastewater samples	HPLC-UV	0.041 μg/L	–	0.70–150.0 μg/L	0.99	74.3–103.8%	<2.75%	[116]
UiO-66-NH_2_	Carbamazepine	Urine and water samples	HPLC	0.05 μg/L	0.13 μg/L	0.1–50 μg/L	0.9988	98.8–99.4%	3.2–2.5%	[117]
MOF-545	Hg^2+^	Fish samples	CVAAS	20 ng/L	–	0.2–50 μg/L	0.989	74.3–98.7%	<3.1%	[118]
Co-MOF	Organic dyes	Seawater samples	HPLC-DAD	0.09–0.38 μg/L	–	0.5–200 μg/L and 1.0–150 μg/L	0.9908–0.999	78.5–99.6%	<6.4%	[119]
Co-MOF	Bisphenol A (BPA)	Juice and drinking water	HPLC-UV	0.07 μg/L	0.3 μg/L	0.3–300 μg/L	–	98.2–99.1%	3.2–5.7%	[120]

**Table 6:** Selected examples of using MOF as sorbents in the MSPD technique.

MOF	Analytes	Matrix/Sample	Analytical Technique	LOD	LOQ	Linearity Range	R^2^	Recovery	RSD	Source
MOFs-MIPs	Three pyrethroids residue	Six wheat samples	GC-MS/MS	1.8–2.8 ng/g	–	10–1000 ng/g	>0.9979	95.7–108.9%	2.7–6.3%	[129]
MIPs-MOFs	Tetracyclines	Milk powder	UHPLC-MS/MS	0.217–0.318 ng/g		1–100 ng/g	>0.998	84.7–93.9%	2.8–7.4%	[130]
Lanthanide-based MOFs	Pesticides	Peppers (*Capsicum annuum L.*)	GC-MS	16.0–67.0 µg/kg	50.3–200.0 µg/kg	50.0–1000.0 µg/kg	>0.9930	48.4–135.0%	6.3–43.6%	[131]
Graphene oxide, MW-CNTs, MOF	Fifteen phthalates	Soil samples (agricultural soil and sand)	UHPLC-MS/MS	–	0.14–2.7 µg/kg	2–101 µg/kg	–	70–120%	<20%	[132]
MOF-808	Five saponins	*(P. ginseng)* leaves	UHPLC-TOF/MS	0.087–0.114 µg/kg	0.292–0.379 µg/kg	0.01–100 µg/mL	>0.998	87.04–103.78%	<5%	[133]
NdMOF LaMOF	Lindane, bifenthrin, pirimicarb	Freeze-dried chicken eggs (*Gallus gallus domesticus*)	GC-MS	0.003–0.015 mg/kg	–	0.01–2 µg/mL	>0.999	70–120%	0.8–7.9%	[134]

**Table 7:** Selected examples of using MOF as sorbents in the SBSE technique.

MOF	Analytes	Matrix/Sample	Analytical Technique	LOD	LOQ	Linearity Range	R^2^	Recovery	RSD	Source
LDH-ZIF-8	Benzyl-penicilin	Biological and food samples	HPLC	0.05 µg/L	0.15 µg/L	0.5–500 µg/L	0.9921	95.0–105.1%	2.8–5.9%	[139]
UiO-66@FGR	Five NSAIDs	Food samples	UHPLC	0.92 ng/mL	–	10–1500 ng/mL		80.8–117.2%	<8.0%	[140]
NH_2_-MIL-101(*Al*)@GMA monolith	Estrone, 17β-estradiol, estriol, 17β-ethinylestradiol	Water and human urine	HPLC-FLD	0.015–0.58 µg/L	0.05–1.9 µg/L	2–750 µg/L	>0.999	70–95%	<6%	[141]
Cu-BDC	Fenthion	Water and fruit samples	Corona discharge IMS	0.1 µg/L	–	0.5–80 µg/L	–	88–111%	6.4–8.6%	[142]
UiO-66-(OH)_2_	Enoxacin, norfloxacin, ciprofloxacin	Fish and shrimp samples	UHPLC	0.48–0.8 ng/mL	–	10–300 ng/mL	>0.9989	74.8–115.8%	<6.9%	[143]

## Data Availability

Data are made available by the authors upon request.
